# Drought resistance of *Argania spinosa* L. colonized by the arbuscular mycorrhizal fungus *Rhizophagus irregularis* varies according to accession

**DOI:** 10.3389/fpls.2025.1678553

**Published:** 2025-10-15

**Authors:** Matike Ganoudi, Soumia El Malahi, Nouhaila Manan, Mohammed Ibriz, Maryline Calonne-Salmon, Stéphane Declerck

**Affiliations:** 1 Biotechnology Research Unit, Center of Agricultural Research of Rabat National Institute of Agricultural Research, Rabat, Morocco; 2 Earth and Life Institute, Applied Microbiology, Mycology, Université Catholique de Louvain, Louvain-la-Neuve, Belgium; 3 Laboratory of Microbial Biotechnology and Plant Protection, Faculty of Sciences, Ibn Zohr University, Agadir, Morocco; 4 Laboratory of Applied Plant Biotechnology, Faculty of Sciences, Mohammed V University, Rabat, Morocco; 5 Laboratory of Plant, Animal and Agro-Industry Production, Faculty of Sciences, Ibn Tofail University, Kenitra, Morocco

**Keywords:** *argania spinosa*, arbuscular mycorrhizal fungi, water stress, phosphorus uptake, antioxidant metabolism, accession variability

## Abstract

*Rhizophagus irregularis* MUCL 41833 on three argan (*Argania spinosa* L. Skeels) accessions (Tidzi, Mejji, and City Hanchan) under well-watered (100% field capacity) and water-stressed (15% field capacity) conditions. Whatever the water regime, AMF colonization was observed in all accessions, but Tidzi showed significantly higher total root colonization than City Hanchan, while Mejji showed intermediate levels. Under well-watered conditions, the colonized plants exhibited higher biomass, root length, chlorophyll content, stomatal conductance, and root potassium (K) concentration in all accession. In the Mejji accession, colonized plants also had significantly higher shoot concentrations of phosphorus (P) and K. Under water-stressed conditions, plant response varied with accession. Compared to their respective controls, Mejii had significantly higher biomass, shoot K concentration, chlorophyll content, stomatal conductance, and reduced oxidative stress, Tidzi had also significantly higher biomass, root P and K concentration, and chlorophyll content with lower oxidative stress, while City Hanchan had significantly higher biomass and root P concentration but had higher H_2_O_2_ concentration. We can conclude that mycorrhization benefits all three accessions under stress conditions, with the Mejji and Tidzi accessions responding more favorably than the City Hanchan accession. These results highlight the role of AMF in improving argan tree performance under water-limiting conditions and demonstrate the variability in response between accessions.

## Graphical Abstract

**Figure:**
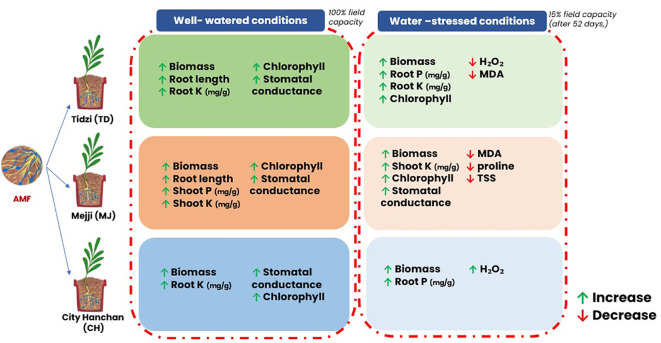

## 1 Introduction

*Argania spinosa* (L.) Skeels is an endemic tree species native to Morocco which is of immense economic and social importance, particularly in the arid and semi-arid regions of the country. It plays a vital role in the local economy, providing valuable products such as argan oil (Ganoudi et al., 2025), which is renowned for its many nutritional, cosmetic, and medicinal properties and uses. Roasted kernels produce culinary oils, while unroasted kernels produce cosmetic oils (Mechqoq et al., 2021; El Oumari et al., 2022; Mouahid et al., 2022).

In Morocco, historical data show a clear trend of increasing temperatures and decreasing rainfall (Ouhamdouch and Bahir, 2017), contributing to more frequent and severe droughts (Abdelmajid et al., 2021; Cherif et al., 2023; Ongoma et al., 2024). These changes particularly affect argan groves, which are sensitive to rainfall variability and rising temperatures, threatening the long-term sustainability of both the ecosystem and local communities (Afi et al., 2024).

A number of studies have identified several physiological and biochemical mechanisms by which argan trees respond to drought stress: leaf water potential and stomatal conductance have been shown to decrease significantly under drought stress, limiting CO2 fixation and thus reducing photosynthetic efficiency (Chakhchar et al., 2016; Mouafik et al., 2022). Drought stress has been reported to affect nutrient uptake leading to nutritional imbalances in plants (Chakhchar et al., 2018a, b). A significant decrease in the hydraulic conductivity of argan roots has also been observed under conditions of increasing drought stress (Chakhchar et al., 2019). This phenomenon can significantly impact plant survival, especially under prolonged drought conditions. Finally, an increase in the production of reactive oxygen species (ROS) has been reported under conditions of drought stress, causing oxidative damage to carbohydrates, protein synthesis, and lipid metabolism, as well as to membranes, resulting in plant cell death (Meslem et al., 2015; Chakhchar et al., 2018b).

The TD, MJ, and CH argan accessions originate from different regions and environments, reflecting diverse ecological backgrounds and considerable phenotypic variability. In addition, they are among the most productive accessions in terms of yield and oil quality. These characteristics make them particularly valuable for assessing intraspecific differences in physiological performance and response to AMF colonization under water stress conditions.

To combat drought stress and support argan tree production, traditional irrigation methods are used. These include, among others, localized irrigation using ramps equipped with drippers (El-Otmani et al., 2015; Wifaya et al., 2018). In addition, genetic diversity within the species means that the trees best adapted to drought conditions can be identified and used in plantations (Msanda et al., 2005; Majourhat et al., 2008; Ait Aabd et al., 2020). Other approaches, such as the use of beneficial soil microorganisms, can also help to improve plant resistance to drought (Dai et al., 2022, Yang et al., 2025). For instance, arbuscular mycorrhizal fungi (AMF) have been reported to help plants resist drought stress through various mechanisms. A study synthesizing data from 187 research papers found that AMF inoculation significantly enhanced plant biomass and nutrient uptake, with greater effects on phosphorus than nitrogen (Wu et al., 2024). Another meta-analysis showed that AMF improved plant photosynthesis and water status under drought stress by enhancing gas exchange and water use efficiency, although the effects varied depending on drought severity (Chandrasekaran, 2024). In wheat, AMF inoculation increased chlorophyll content, the maximum quantum efficiency of PSII, and relative water content (RWC%), while regulating antioxidant enzyme activity, proline levels, and reducing hydrogen peroxide (H_2_O_2_), superoxide (O_2_^−^), electrolyte leakage (EL%), and lipid peroxidation (MDA) under drought stress (Abdelaal et al., 2024). Meta-analyses also highlighted the role of AMF in promoting soil functions, such as aggregation and microbial biomass, enhancing soil multifunctionality and drought resistance (Tang et al., 2024). Overall, AMF improved nutrient uptake, strengthened soil structure, increased water use efficiency, and boosted antioxidant defenses mechanisms, particularly in drought-stressed environments (Das and Sarkar, 2024; Nie et al., 2024; Wang et al., 2024). As a result, inoculating argan trees with AMF to increase their resistance to drought is an option that should be considered, although it is not given much thought at present. Only a few studies have shown that AMF inoculation can improve the growth, mineral nutrition, and physiological parameters of argan plants under drought stress (Ouallal et al., 2022; Outamamat et al., 2022a; Soufiani et al., 2023).

Given the importance of argan tree in arid ecosystems and its vulnerability to water scarcity, it is crucial to evaluate whether AMF can improve its drought tolerance. Therefore, in the present study, we investigated the impact of the AMF *Rhizophagus irregularis* MUCL 41833 on the morphological, physiological, and biochemical parameters, nutrient status, osmolyte accumulation, and antioxidant metabolism responses of three argan accessions grown under two distinct water regimes: well-watered and water- stressed. The aim of the study was to gain a better understanding of some of the mechanisms by which the AMF can increase the resistance of argan trees to drought stress and to explore whether there are differences between accessions, ultimately contributing to the selection of the best accessions for cultivation in water-scarce regions.

## 2 Materials and methods

### 2.1 Biological material

#### 2.1.1 *Argania spinosa* (L.) Skeels

Argan fruits were collected from three different sites in the Essaouira region of Morocco, namely City Hanchan (CH), Mejji (MJ) and Tidzi (TD) (hereafter referred to as argan accessions). The fruits of the TD accession are oval-shaped, those of the CH accession are rounded, while the fruits of the MJ accession are elongated. These accessions were selected for their strong rooting, high productivity, and distinct phenotypic characteristics (see for more details in Ganoudi et al., 2021).

#### 2.1.2 Arbuscular mycorrhizal fungus

*Rhizophagus irregularis* (Błaszk., Wubet, Renker & Buscot) C. Walker & A. Schüßler comb. nov., strain MUCL 41833, was obtained from the Glomeromycota IN vitro COllection (GINCO^1^,^2^). It was cultured in bi-compartmented Petri plates with Ri-T DNA-transformed roots of carrot (*Daucus carota* L.) strain DC2, as described by St-Arnaud et al. (1996) and Cranenbrouck et al. (2005). After three months, thousands of spores were produced in the hyphal compartment (devoid of carrot hairy roots) of the bi-compartmented Petri plates. The spores were used to inoculate maize plants in 1 L pots containing sterilized (twice at 121°C for 15 min) vermiculite and lava stone (w:w, 1:1) to produce the AMF inoculum. After 3 months, root colonization reached 98.5% and tens of thousands of spores were produced.

### 2.2 Experimental design

The experimental design aimed at evaluating the effects of AMF colonization (M treatment) on the drought resistance of three argan accessions (TD, MJ, and CH) grown under two water regimes: well-watered (WW = 100% Field Capacity, FC) and water- stressed (WS = 15% FC). Non-mycorrhizal plants (NM treatment) were produced and grown in a strict similar way.

The plants were first grown in trays (46 x 24 cm) in the greenhouse at a temperature of 25 ± 3°C, 330 µmol m^−^² s^−^¹ light intensity, and a relative humidity (RH) of 60% for 3 months in presence (M) or absence (NM) of inoculum of the AM fungus. They were then transferred to 1 L pots for acclimatization during 50 days. Finally, half of the plants were subjected to a decrease in water content (during 45 days) until reaching 15% of FC and then kept for an additional 7 days under this WS condition, while the other half was kept at 100% FC (WW) during the 52 days. This resulted in four treatments: (1) M/WW, (2) M/WS, (3) NM/WW (4) NM/WS for each accession. (see below for details for the whole process). Each treatment consisted of 12 plants per accession. Plants were randomly placed on two tables in the greenhouse and pots were rotated every two weeks to minimize positional bias.

#### 2.2.1 Seed sterilization, germination, and growth conditions

The experimental process began with the preparation of the argan seeds. The fruits were peeled and shelled to release the almonds, which were surface disinfected with a solution of 8% sodium hypochlorite for 15 minutes. The almonds were then rinsed three times (15 min each) with sterilized (121°C for 15 min) water to ensure the complete removal of the sodium hypochlorite. The almonds were finally placed per two in Petri plates on the Modified Strullu-Romand (MSR) medium (Declerck et al., 1998) for germination. The Petri plates were incubated in the dark at 27°C for 12 days, achieving a germination rate of 81.5%. The Petri plates with germinated almonds were then transferred to a growth chamber with a photoperiod of 16 h day^1^ (24°C/20°C, day/night), a RH of 50%, and a light intensity of 170 µmol m^−^² s^−^¹ for four days.

#### 2.2.2 Mycorrhizal inoculation and greenhouse growth

Nine trays (three trays per argan accession) were prepared, containing 1200 g of sterilized soil (twice at 121°C for 1 hour). The soil sterilization was done to avoid root colonization by the AMF present in the collected soil. The sterilized soil was then left two weeks to allow for escape of volatile phytotoxic/mycotoxic substances produced during the process of heating (Koide and Li, 1989; Mwangi et al., 2011). The sterile soil was mixed with 500 g of substrate containing *R. irregularis* inoculum. The chemical characteristic of the soil is presented in **Table 1**. Nine trays containing the same soil substrate without AMF inoculum were considered for the NM treatments.

**Table 1:** Chemical properties of soil samples from each site and from the greenhouse.

Sites	pH	Organic matter (%)	P_2_O_5_ (ppm)	K_2_O (ppm)	CE (mS/Cm)
TD	8.26	4.9	0.8	295.2	1.6
MJ	8.03	1.8	2.4	1000.2	4.4
CH	8.17	8.7	145.0	102.4	1.5
Greenhouse soil	8.10	1.2	18.6	168.7	0.6

Sixteen-days-old argan plants, with a primary root of 2 to 10 cm, were transferred into the trays (i.e., 16 plants per tray – 3 trays per accession) and were maintained under controlled greenhouse conditions (temperature of 25 ± 3°C, light intensity of 330 µmol m^−^² s^−^¹ and a RH of 60%) for three months. Plants were initially watered with distilled water and then every three days with 1 L of Hoagland’s solution (Hoagland and Arnon, 1950). At the end of this phase, root colonization was assessed on three randomly selected M and NM plants for each accession. The total root colonization reached 86.2% ± 7.3%, 79% ± 2.1%, and 75.7% ± 8% for TD, MJ, and CH, respectively, while no root colonization was detected in the plants of the NM treatments.

#### 2.2.3 Plants acclimatization

Among the 48 M and NM argan plants per accession in total, 36 were selected based on uniformity of size and number of leaves and transferred into 1 L pots (one plant per pot) containing a sterilized (twice at 120°C for 1 h) mixture of soil collected from the field site of each accession and compost (2/3 soil, 1/3 potting soil (Plantaflor, European Union), v/v).The pot cultivation ensured uniform growth conditions, but their limited size imposed physical confinement to the root system, potentially limiting root/AMF exploration, thus potentially influencing nutrient acquisition and the magnitude of AMF benefits. The chemical characteristics of the soils from each site are presented in **Table 1**. The plants were acclimatized in the greenhouse for 50 days at a temperature of 25 ± 3°C, a light intensity of 330 µmol m^−^² s^−^¹, and a RH of 60%. All plants were irrigated to maintain 100% FC to ensure uniform development prior to stress application.

The calculation of FC was as follows: The different soil/compost substrates were saturated by adding distilled water until puddling occurred. After 24–48 hours of free drainage in a controlled environment at 24°C, the wet weight (ww) of the substrates were determined. They were then oven dried at 105°C for 24 hours to obtain the dry weight (dw). The FC was calculated using the formula: FC (%) = ww-dw*100/dw.

#### 2.2.4 Soil moisture management using weight-based soil drying method

After the acclimatization phase, 24 plants of uniform size were selected from the 36 acclimated plants in each treatment (M or NM) and accession to apply the water regimes. The selected plants were then divided into WW and WS conditions, constituting twelve conditions as follows: TD^M/WW^, TD^M/WS^, TD^NM/WW^, TD^NM/WS^, MJ^M/WW^, MJ^M/WS^, MJ^NM/WW^, MJ^NM/WS^, CH^M/WW^, CH^M/WS^, CH^NM/WW^, CH^NM/WS^. Twelve plants were considered per treatment and randomly distributed in the greenhouse. Pot rotation was performed every two weeks to minimize positional bias. Temperature, RH, and photoperiod were kept constant throughout the experiment, as described earlier.

Soil moisture levels were controlled using a precise weight-based soil drying method, using a high precision balance (Bionlock, BS 3000). This approach involved monitoring the combined weight of the pot, soil, and plant daily to maintain target moisture levels corresponding to specific FC. For the plants of the WW treatment, pots were initially weighed at full FC, and water was added as required to maintain this level. For the plants of the WS treatment, plants were not watered until pots reached 15% of their FC (after 45 days) This FC was kept for an additional 7 days by controlled irrigation.

### 2.3 Plant harvest

At the end of the experiment (i.e., 52 days after initiating the two water regimes), plants were harvested and root colonization, plant height and number of leaves, mineral nutrient content (phosphorus (P) potassium (K) and sodium (Na)), physiological (relative water content (RWC), chlorophyll content, and stomatal conductance (g_s_)), and biochemical (total soluble sugars (TSS**),** proline content, malondialdehyde (MDA) and hydrogen peroxide content (H_2_O_2_)) parameters were measured (see sections 2.4, 2.5, 2.6, 2.7 and 2.8). In each treatment, twelve plants were used to assess height and number of leaves. From these 12 plants, three were further used to determine root colonization, three for fresh and dry weights and mineral nutrient content, three for physiological parameters and three for biochemical parameters.

### 2.4 Assessment of the root colonization

The percentage of root length colonized (%CRL) by the AMF was estimated following the method of Philips and Hayman (1970). Roots were carefully harvested, thoroughly washed-free from debris, and cleared with 10% KOH. After neutralization with 1% HCl for three min, the roots were bleached in a solution of hydrogen peroxide (H_2_O_2_) 3.5% at 90°C for 10 min in a water bath. The roots were then stained with 2% CROWN Blue Ink (PRC) in 1% HCl (Walker, 2005) by placing the tubes in a water bath at 70°C for 30 min. Root colonization was quantified using optical microscopy (Olympus CX33RTFS2, Hamburg, Germany, version 2024) at 40X magnification (McGonigle et al., 1990). Three slides with 30 1-cm-long root fragments were made per plant to estimate the percent colonization using the line-intercept method, noting hyphae, arbuscules and spores/vesicles. For each plant, between 100 and 150 intercepts were observed (McGonigle et al., 1990).

### 2.5 Evaluation of plant height, number of leaves and weights

Shoot height and number of leaves were measured. Shoot height was measured from the base of the stem to the crossing point of the last leaf. Shoot and root fresh weights (SFW and RFW, respectively) were determined. Shoots and roots dry weights (SDW and RDW, respectively) were determined after drying in an oven at 65°C for 72h, until a constant weight was reached.

### 2.6 Determination of mineral nutrient

Phosphorus, potassium, and sodium concentrations were determined in fresh leaves, oven-dried at 60-70°C for 48 hours, and then incinerated at 500°C for 5 hours. The P concentration was registered using a METASH 5100 model UV/VIS spectrophotometer at a wavelength of 825 nm. Potassium and Na concentrations were measured using a flame photometer (BWB-XP Technologies UK LTD), according to the method described by Havre (1961). Available P (P_2_O_5_) was determined using the Olsen method (Olsen and Sommers, 1982), with sodium bicarbonate extraction performed at pH 8.5. Available P (P_2_O_5_) was recorded using a JENWAY 6405 UV-visible spectrophotometer.

### 2.7 Physiological parameter assessment

#### 2.7.1 Relative water content

Relative water content (RWC) was measured on five whole leaves per plant. The RWC was calculated as RWC = (FW−DW)/(TW−DW) × 100, where FW and DW are the fresh and dry (drying at 70°C for 48h) weights, respectively, and TW is the turgid weight after leaves were soaked in distilled water for 24h at 4°C in the dark.

#### 2.7.2 Stomatal conductance (gs)

Stomatal conductance (gs) was measured using a leaf porometer (SC-1 leaf porometer, version 2014, Decagon Devices, USA) between 10:00 and 12:00 AM. Data were collected from the same leaf located at the center of the plant.

#### 2.7.3 Chlorophyll content

Chlorophyll content was measured using a SPAD-502 portable chlorophyll analyzer developed by Soil and Plant Analysis Developments (KONICA MINOLTA, Japan). The SPAD-502 is a widely used, handheld instrument designed for the rapid and precise assessment of leaf chlorophyll content. It functions by measuring the light transmitted through the leaf in the red and near-infrared wavelengths (700 nm–780 nm) (Shibaeva et al., 2020). Prior to taking measurements, the SPAD meter was calibrated using the manufacturer’s reference checker. The SPAD value for each leaf was determined as the average of 6 readings. The meter provides a digital reading between 0.0 and 99.9 SPAD units, which corresponds to chlorophyll content.

### 2.8 Biochemical parameter evaluation

#### 2.8.1 Total soluble sugars

Fresh leaves (500 mg) were dried at 60°for 48h, extracted with 5 mL of 70% ethanol and centrifuged at 4000 rpm for 10 min. Extraction was repeated 2 times with 70% ethanol and supernatants were collected into 25 mL volumetric flasks. The extract (0.5 mL) was pipetted from each treatment into separate test tubes containing 0.5 mL of 5% phenol, followed by 2.5 mL of concentrated sulfuric acid (90%). The mixture was then incubated for 30 minutes on ice in the dark to ensure reaction stability. The intensity of color was read at 490 nm using a spectrophotometer (GENESYS 10S UV. VIS). A standard curve was prepared using 1g of glucose per 100 mL of distilled water. Total soluble sugar (mg/g) = Sample O.D x standard concentration (mg/mL) x dilution factor/standard O.D x sample weight (g).

#### 2.8.2 Proline concentration

The proline concentration was assessed according to the procedure of Carillo and Gibon (2011). Fifty mg of leaf sample was weighed and then extracted using ethanol (500 µL of 80% (v/v) heated at 80°C for 20 min). One hundred µL of a solution consisting of 1% (w/v) ninhydrin, 20% absolute ethanol (v/v), 60% glacial acetic acid (v/v), and 20% ultrapure water was added to 75 µL supernatant in the 96 wells microplate. The microplate was boiled at 80°C for 30 min. After cooling at room temperature, the absorbance was recorded at 520 nm using a microplate reader. Proline concentration was calculated from a calibration curve using proline as standard (Carillo and Gibon, 2011).

#### 2.8.3 Malondialdehyde and hydrogen peroxide concentrations

The extraction was performed by 5 mL of 5% (w/v) trichloroacetic acid (TCA) per 0.20 g of tissue powder. After extraction in the ice bath, homogenates were centrifuged for 10 min at 12,000× g at 4°C and supernatants were used for determination of lipid peroxidation and hydrogen peroxide.

Lipid peroxidation was measured as concentration of malondialdehyde (MDA) in the leaves by the method of Verma and Dubey (2003). A total of 2 mL of the supernatant was mixed with 2 mL of 0.67% thiobarbituric acid (TBA) in 20% trichloroacetic acid (TCA) and was thereafter heated for 30 min at 95°C in a heating block and then cooled on ice. After centrifugation for 1 min at 12,000× g at 4°C, the absorbance of the supernatant was read at 532 and 600 nm. The concentration of MDA was estimated by using the extinction coefficient of 155 mM^−1^ cm^−1^, and concentration was expressed as nanomole per gram of fresh weight (nmol g^-1^ FW).

Hydrogen peroxide (H_2_O_2_) concentration was quantified according to Velikova et al. (2000). After the addition of 0.5 mL of supernatant into 10 mM potassium phosphate buffer (pH 7.0) (0.5 mL) and 1 M potassium iodide (1 mL), reaction mixture was stored in the dark for 20 min. Absorbance of the reaction mixture was read at 390 nm, and H_2_O_2_ concentration was determined using a calibration curve obtained with different concentrations of H_2_O_2_ and expressed as µmole per gram of fresh weight (µmol g^-1^ FW).

### 2.9 Statistical analysis

Treatment effects were analyzed using analysis of variance (ANOVA) at a 95% confidence level. All analyses were conducted in Minitab^®^ version 20 (Minitab, Inc., State College, PA, USA). Data were first checked for normality using the Shapiro–Wilk test and for homogeneity of variances using Levene’s test. For datasets meeting both assumptions, one-way ANOVA was applied, followed by Tukey’s *post hoc* (HSD) test for pairwise comparisons. Tukey’s HSD inherently adjusts for the number of comparisons, thereby controlling the family-wise error rate and minimizing Type I error inflation.

When normality or variance assumptions were not satisfied, the Kruskal–Wallis test was employed as a non-parametric alternative. In cases of significant Kruskal–Wallis results, pairwise comparisons between treatments were performed using Dunn’s test with Bonferroni correction to account for multiple testing and control Type I error. The selection of parametric versus non-parametric procedures was based strictly on the distributional properties of each parameter. Specifically, ANOVA was used to analyze root length, shoot height, number of leaves, fresh and dry weight of roots and shoots, and concentrations of P, K, and Na, as well as %CRL. Conversely, chlorophyll content, gs, RWC, and biochemical parameters were evaluated with the Kruskal–Wallis test.

## 3 Results

### 3.1 Root colonization

**Figure 1:**
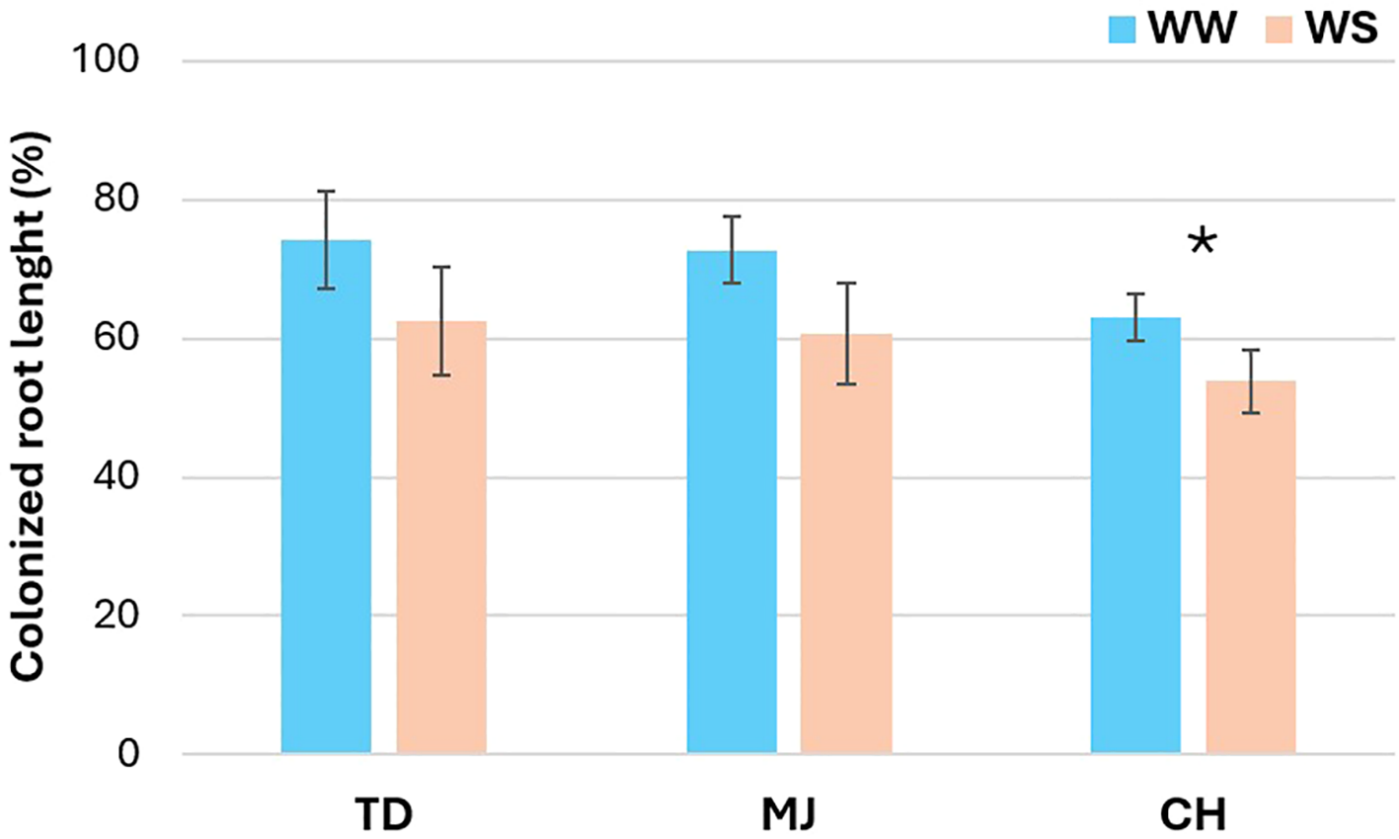
Effect of water regime (WR) (i.e., 100% of field capacity= Well-Watered (WW) and 15% of field capacity = Water-Stressed (WS)) on the percentage of colonized root length (%CRL) by *Rhizophagus irregularis* MUCL 41833 of three argan accessions (i.e., TD, MJ, and CH). Data are presented as means ± standard deviation. The presence of * indicates a significant difference among the means between WW and WS treatments for each accession, according to a one-way ANOVA followed by a Tukey’s HSD *post-hoc* test (P ≤ 0.05).

Root colonization was measured at harvest, i.e., after the application of the two WR (WW and WS) during 52 days. Whatever the WR, a significant higher %CRL was noticed for the TD accession (74.1 ± 7.0% and 62.4 ± 7.8%, for WW and WS, respectively) compared to the CH accession (63.0 ± 3.4% and 53.8 ± 4.5%, for WW and WS, respectively), while no significant difference was measured between these two accessions and the MJ accession (72.7 ± 4.8% and 60.7 ± 7.3%, for WW and WS, respectively). The %CRL decreased significantly by 14.6% between the WW and WS treatments only for the CH accession, while no significant differences were observed between these two WR for TD and MJ accessions (**Figure 1**).

### 3.2 Plants growth parameters

**Figure 2:**
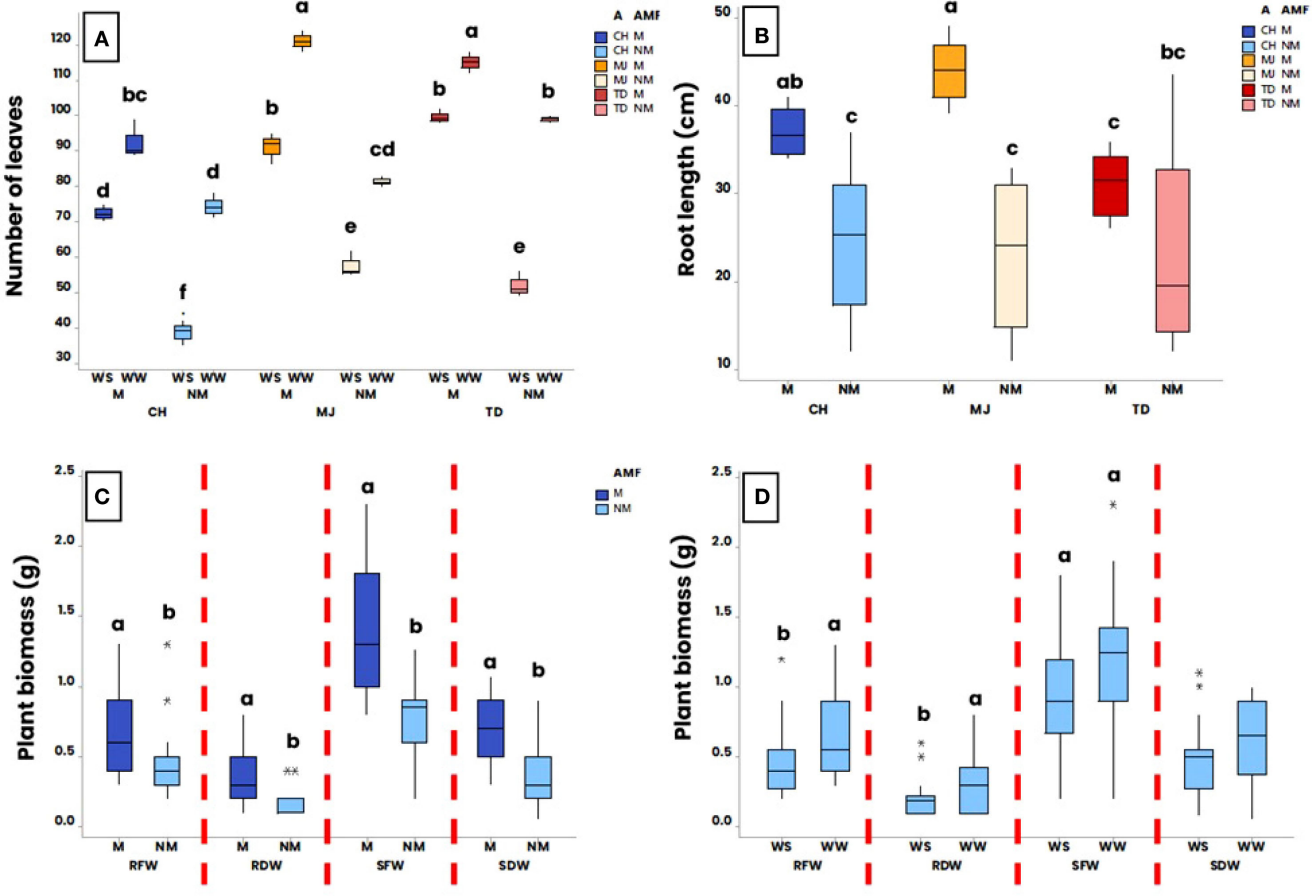
Effect of water regime (WR) (i.e., 100% of field capacity = Well-Watered (WW) and 15% of field capacity = Water-Stressed (WS)), AMF colonization by *Rhizophagus irregularis* MUCL 41833 (i.e. mycorrhizal (M) or non-mycorrhizal (NM)) and accession (i.e., TD, MJ and CH) on plant growth parameters at harvest (52 days after the start of the WR application). Significance of **(A)** accession, AMF and WR interaction on the number of leaves, **(B)** accession and AMF interaction on root length, **(C)** AMF on total biomass (root and shoot fresh and dry weights (RFW, RDW, SFW, SDW)) and **(D)** WR on total biomass. The box plots display the maximum, upper quartile, and lower quartile minimum values. Data were analyzed by a three-way ANOVA followed by a Tukey *post-hoc* test (P ≤ 0.05). For one parameter, data sharing similar lower-case letters are not significantly different. (*) indicates an extreme point (> 1.5 times the interquartile range beyond the quartiles), while (**) denote a very extreme point (> 3 times the interquartile range).

At harvest, a significant effect of the interaction “accession x AMF x WR” (p ≤ 0.001) was measured on the number of leaves. Whatever the accession and WR, a significant higher number of leaves was observed in the M plants compared to the NM plants (**Figure 2A**). Similarly, whatever the accession and presence/absence of AMF, a significant higher number of leaves was noticed for plants grown in the WW condition compared to the WS condition (**Figure 2A**).

A significant effect of the interaction “AMF x WR” was observed on shoot length (p ≤ 0.01) (results not shown). Averaged over the accessions, the shoot length of the NM plants was significantly lower in the WS condition (13.0 ± 2.8 cm) compared to the WW condition (19.7 ± 4.1 cm), while no significant difference was observed between M plants grown under WW (23.6 ± 3.0 cm) and WS (23.4 ± 2.7 cm) conditions. Under WW conditions, no significant difference in shoot length was observed between M and NM plants, whatever the accession. However, under WS conditions, M plants had significantly longer shoots than NM plants.

A significant effect of the interaction “accession x AMF” and “AMF x WR” (p ≤ 0.01 and p ≤ 0.001, respectively) was observed on root length. Averaged over the two WR, the root length was significantly higher in M plants of the CH and MJ accessions compared to their respective NM plants, while no significant difference was observed for TD accession (**Figure 2B**). Averaged over the accessions, no significant difference was observed in root length between M plants grown under WW (36.8 ± 2.3 cm) and those grown under WS (37.8 ± 7.0 cm) conditions (results not shown). In contrast, for the NM plants, the root length was significantly lower under WS conditions (16.8 ± 4.8 cm) compared to WW conditions (30.3 ± 7.4 cm). Similar to shoot length, no significant difference in root length was observed between M and NM plants under WW conditions, regardless of accession, while under WS conditions, M plants had significantly longer roots than NM plants.

Plant biomasses (i.e., SFW, SDW, RFW and RDW) were significantly impacted by the factor “AMF” (p ≤ 0.001, p ≤ 0.001, p ≤ 0.01 and p ≤ 0.001 respectively). Averaged over both the accession and the WR, the SFW, SDW, RFW and RDW were significantly higher for the M plants compared to the NM plants (**Figure 2C**). Except for SDW (p = 0.06), the factor “WR” also influenced the SFW, RFW and RDW (p = 0.02, p = 0.02 and p = 0.03, respectively). Averaged over the accessions and the presence/absence of AMF, no significant difference was observed in SFW and SDW between plants grown under WW and WS conditions, while RFW and RDW were significantly lower under WS conditions (**Figure 2D**). No significant effect of the interaction “AMF x WR” was observed on SFW, SDW, RFW and RDW (p = 0.5, p = 0.2, p = 0.3 and p = 0.3, respectively).

### 3.3 Plant nutrient concentrations

**Figure 3:**
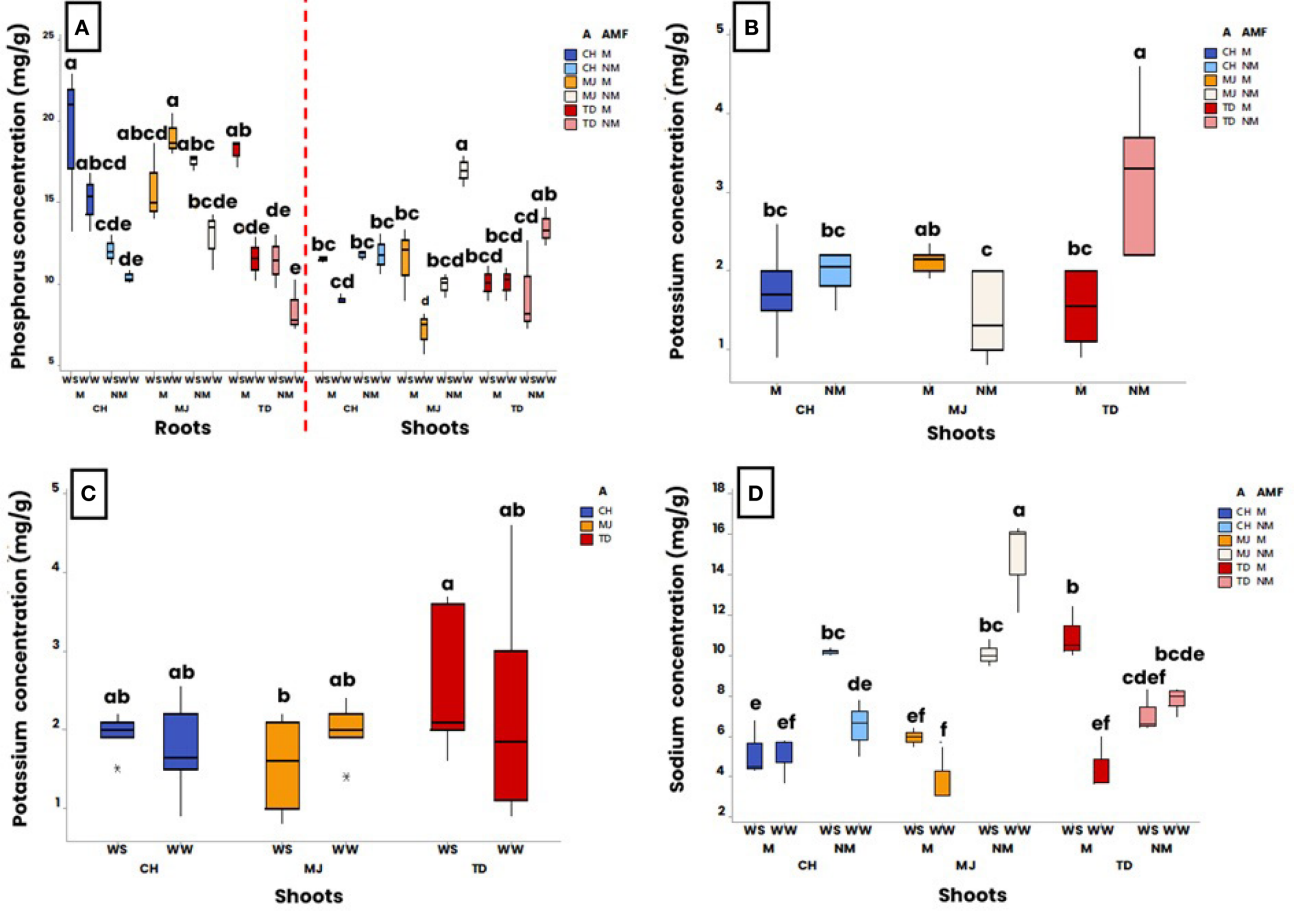
Effect of water regime (WR) (i.e., 100% of field capacity = Well-Watered (WW) and 15% of field capacity = Water-Stressed (WS)), AMF colonization by *Rhizophagus irregularis* MUCL 41833 (i.e. mycorrhizal (M) or non-mycorrhizal (NM)) and accession (i.e., TD, MJ, and CH) on nutrients concentrations at harvest (52 days after the start of the WR application). Significance of **(A)** accession, AMF and WR interaction on phosphorus concentration **(B)** accession and AMF interaction on potassium concentrations in shoots, **(C)** accession and WR interactions on potassium concentrations in shoots and **(D)** accession, AMF and WR interaction on sodium concentrations in shoots. The box plots display the maximum, upper quartile, and lower quartile minimum values. Data were analyzed by a three-way ANOVA followed by a Tukey *post-hoc* test (P ≤ 0.05). For one parameter, data sharing similar lower-case letters are not significantly different.

At harvest, P, K and Na concentrations were measured in roots and shoots (**Figure 3**).

#### 3.3.1 Phosphorus dynamic under water conditions

Phosphorus concentration was impacted by the interaction “accession x AMF x WR” in both roots (p ≤ 0.001) and shoots (p ≤ 0.01). In roots, no significant difference in P concentration was measured between plants grown under WW conditions and those grown under WS conditions for M and NM plants in the CH and MJ accessions, as well as for NM plants in the TD accession (**Figure 3A** left). In contrast, a significant increase in P concentration was observed in M plants of the TD accession grown under WS conditions in comparison with those grown under WW conditions. Under WS conditions, a significant difference in P concentration was observed between M and NM plants of the CH and TD accessions, whereas no significant difference in P concentration was observed between M and NM plants of these accessions under WW conditions. No significant difference in P concentration was observed between M and NM plants of the MJ accession under WS conditions, whereas NM plants presented a lower P concentration than the M plants under WW conditions.

In shoots, no significant difference in P concentration was measured between M and NM plants grown under WW and WS conditions in the CH accession (**Figure 3A** right). In contrast, a significant difference in P concentration was observed between plants grown under WW and WS conditions in the MJ accession. For the TD accession, no significant difference in P concentration was found between M plants grown under WW and those grown under WS conditions, whereas NM plants grown under WS conditions had a lower P concentration than NM plants grown under WW conditions. Whatever the WR, no significant difference was observed between M and NM plants in the TD and CH accessions. In the MJ accession, the P concentration in the shoots of M plants grown under WW conditions was significantly lower than in those grown under WS conditions, whereas in the NM plants, the P concentration under WW conditions was significantly higher than in those grown under WS conditions.

#### 3.3.2 Potassium dynamic under water conditions

The K concentration in roots was influenced by the factor “AMF” (p ≤ 0.01). Averaged over both the accession and WR, K concentration was higher in M plants (1.7 ± 0.4 mg/g) than in NM plants (1.2 ± 0.3 mg/g) (results not shown). In shoots, K concentration was impacted by the interactions “accession × AMF” (p ≤ 0.001), “accession x WR” (p ≤ 0.05), and “AMF x WR” (p ≤ 0.05). Averaged over the two WR, no significant difference in K concentration was observed between M and NM plants in the CH accession (**Figure 3B**). Conversely, whereas the M plants had significantly higher K concentrations than NM plants in the MJ accession, the opposite was observed in the TD accession. Averaged over the accessions, no significant difference in K concentration in shoot was observed between WW (1.0 ± 0.2 mg/g) and WS (1.1 ± 0.1 mg/g) conditions for the M plants. Similarly, no significant difference in K concentration was observed between WW (1.4 ± 0.2 mg/g) and WS (0.9 ± 0.2 mg/g) conditions for the NM plants (results not shown). On the other hand, whereas shoot of M plants presented a lower K concentration than the NM plants, the shoot K concentration remained similar between M and NM plants grown under WS conditions. Averaged over the M and NM plants, no significant difference in shoot K concentration was observed between plants grown under WW and WS conditions, irrespective of the accession (**Figure 3C**). On the other hand, whereas plants grown under WW conditions presented similar K concentration, a significantly higher K concentration was measured in the TD accession in comparison with plants of the MJ accession, under WS conditions.

#### 3.3.3 Sodium dynamic under water conditions

The shoot Na concentration was impacted by the interaction “accession x AMF x WR” (p ≤ 0.001). No significant difference in Na concentration was observed between M plants grown under WW and WS conditions in the CH and MJ accessions (**Figure 3D**). Conversely, a significant higher Na concentration was observed in the shoots of M plants grown under WS conditions in the TD accession. In addition, a significant difference in Na concentration was observed between NM plants grown under WW and WS conditions in the CH and MJ accessions, while it remained similar for the TD accession. In the CH accession, Na concentration was higher in NM plants grown under WS conditions than in those grown under WW conditions, while the opposite was observed in the MJ accession.

In the roots, Na concentration was influenced by the factor “WR” (p ≤ 0.001) and the interaction “accession x AMF” (p ≤ 0.001). Averaged over both the accessions and the presence/absence of AMF, the Na concentration was significantly higher in roots of plants grown under WS conditions (8.2 ± 2.5 mg/g) than in those grown under WW conditions (7.09 ± 4.0 mg/g) (results not shown). Averaged over the WR, no significant difference was observed in Na concentration between M and NM plants for all accessions.

### 3.4 Physiological parameters

**Figure 4:**
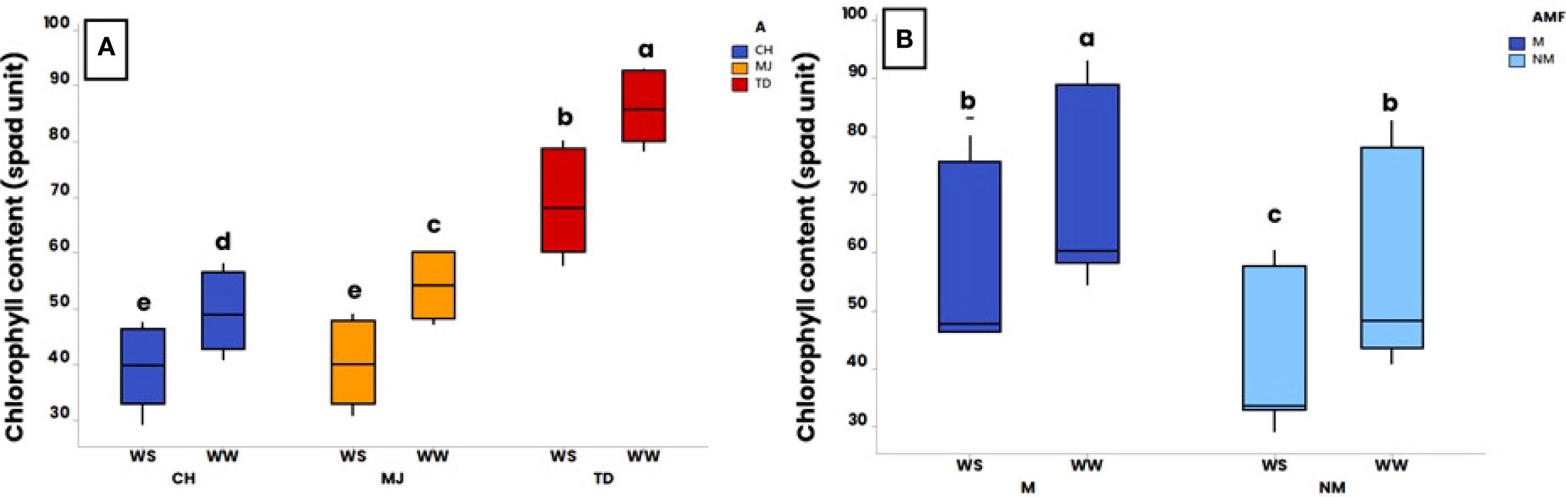
Effect of water regime (WR) (i.e., 100% of field capacity = Well-Watered (WW) and 15% of field capacity = Water-Stressed (WS)), AMF colonization by *Rhizophagus irregularis* MUCL 41833 (i.e. mycorrhizal (M) or non-mycorrhizal (NM)) and accession (i.e., TD (Tidzi), MJ (Mejji), and CH (City El Hanchan)) on chlorophyll content at harvest (52 days after the start of the WR application). Significance of **(A)** accession and WR interaction on chlorophyll content, **(B)** AMF and WR interaction on chlorophyll content. The box plots display the maximum, upper quartile, and lower quartile minimum values. Data were analyzed by a three-way ANOVA followed by a Tukey *post-hoc* test (P ≤ 0.05). For one parameter, data sharing similar lower-case letters are not significantly different.

At harvest, plant physiological parameters (i.e., Chl, g_s_, and RWC) were measured in plants (**Figure 4**).

A significant effect of the interaction “accession x WR” (p ≤ 0.001) and “AMF x WR” (p ≤ 0.01) was observed for Chl content. Averaged over the presence/absence of AMF, Chl content was significantly higher in plants grown under WW conditions compared to those grown under WS conditions for all the accessions (**Figure 4A**). In addition, whatever the WR, the Chl content in accession TD was significantly higher than in MJ and CH accessions. Averaged over the accessions, the Chl content was significantly higher in the M plants than in NM plants, in both WW and WS conditions (**Figure 4B**). In both the M and NM plants, the Chl content was also significantly higher in the plants grown under WW conditions compared to those grown under WS conditions.

The parameter g_s_ was impacted by the factor “AMF” and the factor “WR” (p ≤ 0.01 for each). Averaged over both the accession and the WR, g_s_ was significantly higher in the M plants (262.5 ± 25.5 mmol m^-2^ s^-1^) compared to the NM plants (150.4 ± 38.7 mmol m^-2^ s^-1^) (results not shown) and averaged over both the accession and the presence/absence of AMF, plants grown under WW conditions had significantly higher g_s_ (235.5 ± 50.7 mmol m^-2^ s^-1^) than those grown under WS conditions (177.4 ± 66.6 mmol m^-2^ s^-1^) (results not shown).

The RWC was significantly influenced by the factor “WR” (p ≤ 0.01). Averaged over both the accession and the presence/absence of AMF, plants grown under WW conditions had the highest RWC (89.6 ± 3.7%) compared to those grown under WS conditions (78.9 ± 10.3%) (results not shown).

### 3.5 Biochemical parameters

**Figure 5:**
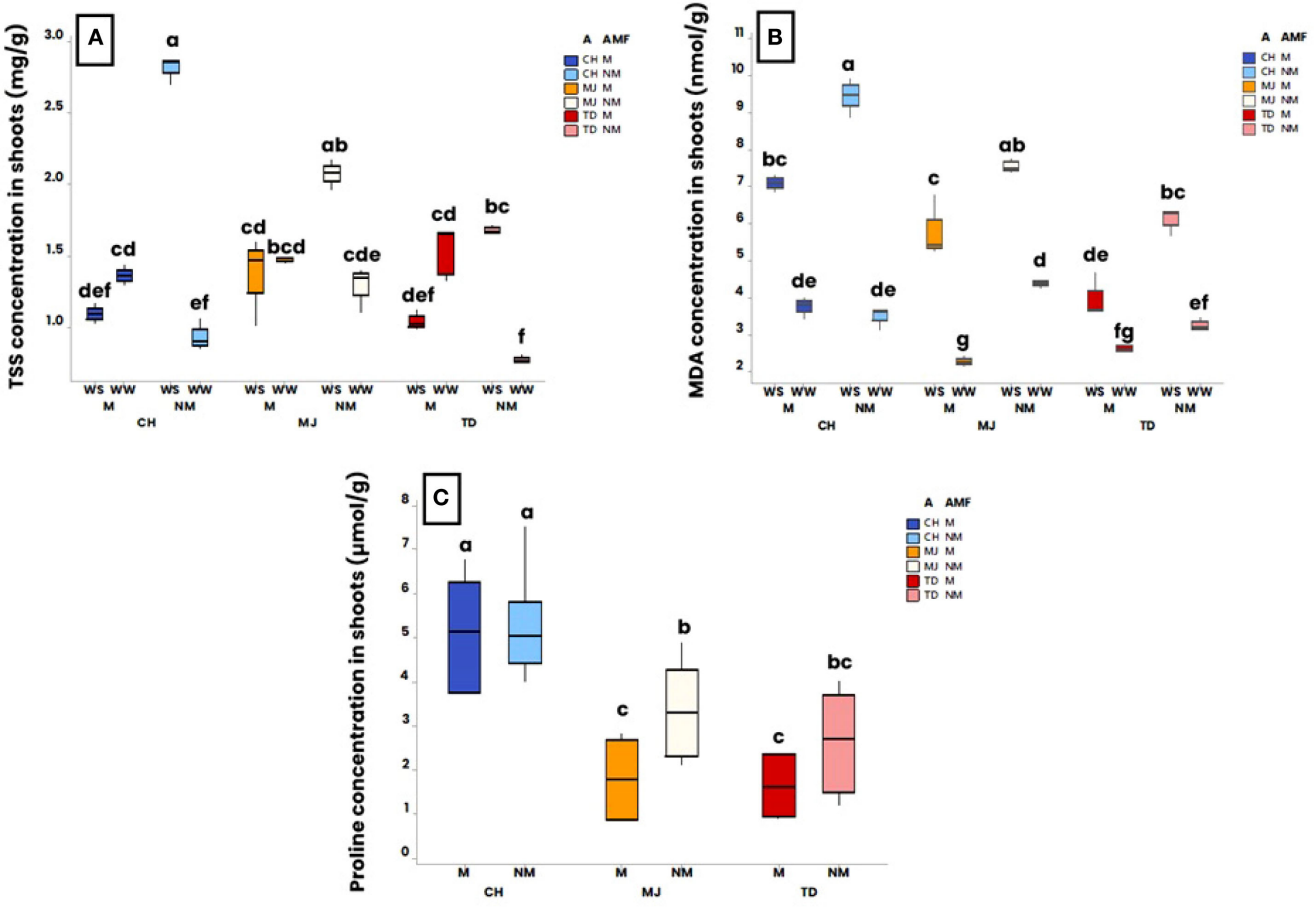
Effect of water regime (WR) (i.e., 100% of field capacity= Well-Watered (WW) and 15% of field capacity = Water-Stressed (WS)), AMF colonization by *Rhizophagus irregularis* MUCL 41833 (i.e. mycorrhizal (M) or non-mycorrhizal (NM)) and accession (i.e., TD, MJ, and CH) on biochemical parameters at harvest (52 days after the start of the water regime application). Significance of **(A)** accession, AMF and WR interaction on TSS concentration, **(B)** accession, AMF and WR interaction on malondialdehyde (MDA), and **(C)** accession and AMF interaction on proline concentration. The box plots display the maximum, upper quartile, and lower quartile minimum values. Data were analyzed by a three-way ANOVA followed by a Tukey *post-hoc* test (P ≤ 0.05). For one parameter, data sharing similar lower-case letters are not significantly different.

At harvest, TSS, MDA, proline concentration and H_2_O_2_ concentrations were measured in the shoots of plants (**Figure 5**).

The TSS was impacted by the interaction “accession x AMF x WR” (p ≤ 0.01). No significant difference in TSS concentration was measured in the M plants grown under WW and WS condition, while in the NM plants, the TSS concentration was significantly higher under WS conditions compared to the WW conditions, irrespective of the accession (**Figure 5A**).

The MDA concentration was also impacted by the interaction “accession x AMF x WR” (p ≤ 0.001). A significantly higher MDA concentration was measured under the WS condition compared to the WW condition, irrespective of the accession and the presence/absence of AMF (**Figure 5B**). In addition, under WS conditions, MDA concentration in shoots was significantly lower in M plants than in NM plants.

Proline concentration was impacted by both the factor “WR” and the interaction “accession x AMF” (p ≤ 0.001 and p ≤ 0.05, respectively). Averaged over both the accession and the presence/absence of AMF, plants grown under WS conditions had a significantly higher proline concentration (4.2 ± 1.6 µmol/g) than those grown under WW conditions (2.4 ± 1.6 µmol/g) (results not shown). Averaged over the two WR, no significant difference was observed in proline concentration between the M and NM plants of the CH and TD accessions, while in MJ, a significant higher proline concentration was observed in the NM plants compared to the M plants (**Figure 5C**). The proline concentration was significantly higher in the M and NM plants of the CH accession in comparison with the accessions MJ and TD.

The hydrogen peroxide concentration in shoot was impacted by the factor “accession”, “AMF” and “WR” (p ≤ 0.001 for each). Averaged over both the presence/absence of AMF and the WR, the CH accession presented significantly higher H_2_O_2_ concentration (2.7 ± 1.5 µmol/g) than MJ (1.9 ± 0.9 µmol/g) which also presented a significantly higher H_2_O_2_ concentration than TD (1.6 ± 0.8 µmol/g) (result not shown). Averaged over both the accession and the WR, NM plants had significantly higher H_2_O_2_ concentration (2.9 ± 1.3 µmol/g) than M plants (1.7 ± 0.9 µmol/g) (result not shown). Finally, averaged over both the accession and the presence/absence of AMF, plants grown under WS conditions had significantly higher H_2_O_2_ concentration (3.0 ± 1.0 µmol/g) than those grown under WW (1.1 ± 0.3 µmol/g) conditions (result not shown).

## 4 Discussion

Drought is one of the main environmental threats affecting plant growth and productivity. The role of AMF in mitigating the effects of this factor on plants is well established (Begum et al., 2019; Bahadur et al., 2019; Diagne et al., 2020; Al-Karaki and Williams, 2021) and has been reported on argan (Outamamat et al., 2022a, 2022b). However, it is not known whether the response of this tree to AMF under drought conditions varies according to accession. Here, we investigated the effects of the AMF *Rhizophagus irregularis* MUCL 41833 on growth, nutrient concentrations, physiological parameters, and biochemical responses of three argan accessions (Tidzi, Mejji, and City Hanchan), grown under two water regimes (WR): well-watered (WW) and water-stressed (WS).

### 4.1 AMF positively impact argan accessions under well-watered conditions

All accessions were colonized, with TD and MJ showing significantly higher root colonization than CH, which correlated with a significant increase in leaf number, root length, and biomasses compared to non-colonized plants. Root length was also significantly increased in the accessions CH and MJ, confirming the often-observed role of AMF in promoting root system development (Ruiz-Lozano, 2003). However, this was not accompanied by a significant difference in shoots or roots P concentration between AMF-colonized and non-colonized plants in the CH and TD accessions, whereas in the MJ accession, P concentration was significantly lower in the shoots and higher in the roots of AMF-colonized plants. The absence of effect on shoot P concentration could reflect dilution, as suggested by Smith et al. (2011), given that colonized plants from all accessions had significantly higher biomass. Though, this was not confirmed in our study; the total P content in shoots did not differ between the AMF-colonized plants and the non-colonized ones (see **Supplementary Results**). This lack of differences may be linked to the form of P and the size of the pots. In fact, P was applied in the form of KH_2_PO_4_ in Hoagland’s solution (1 L every three days) in small pots (1 L), so in a form that was easily available to the roots and in a reduced exploration volume. This probably reduced the added value of hyphae that are able to recover P from less accessible forms or beyond the root depletion zone. Other factors have also been suggested to explain differences between plants, such as AMF species/strain, with some plant/AMF combinations being more efficient than others (Klironomos, 2003; Smith et al., 2011), but these were not explored in the present study.

The AMF-colonized plants had a significantly higher K concentration in roots, irrespective of accession. This suggests that *R. irregularis* MUCL 41833 can improve the uptake of this nutrient via its extraradical hyphae and store it in the intraradical fungal structures and/or root cells. This observation is in agreement with the study by Scheloske et al. (2004), showing a higher concentration of K in *Aster tripolium* roots colonized by *R. irregularis* compared with non-colonized plants. Regarding the shoot K concentration, the plant response differed with accessions. In accession MJ, the AMF-colonized plants had a significantly higher K concentration than the non-colonized plants as previously shown for *Pelargonium peltatum* (Perner et al., 2007) and *Lactuca sativa* (Baslam et al., 2013), while in accession CH, there was no significant difference and in accession TD, it was significantly lower than in the non-colonized plants. This demonstrates, once again, that the effects of AMF may vary depending on plant genotype and host compatibility (Klironomos, 2003; Smith and Smith, 2011). Regardless of the accession, the concentration of Na in the shoots and roots did not differ between AMF-colonized and non-colonized plants. The presence of AMF probably did not influence Na concentration, as plants are probably able to maintain ion homeostasis through their own regulatory mechanisms when water is not a limiting factor (Smith and Read, 2008).

Irrespective of the accession, the chlorophyll content and stomatal conductance of AMF-colonized plants were significantly higher than those of their respective controls. Similar results were obtained by Outamamat et al. (2022a, 2022b). This suggests a better photosynthetic efficiency and improved gas exchange regulation in AMF-colonized plants, as previously documented in other crops such as *Catalpa bungei* (Chen et al., 2020), wheat (Mathur et al., 2019) and hybrid poplar (Liu et al., 2015).

Oxidative stress was reduced in plants colonized by AMF in all accessions in absence of stress. A significantly lower concentration of MDA and H_2_O_2_ was observed in AMF-colonized plants compared with the non-colonized plants. This suggests that the symbiotic relationship enhances the plant’s ability to mitigate oxidative damage, probably by activating antioxidant defense systems and stabilizing cellular structures. It has been shown in the literature that lower MDA concentrations reflect reduced lipid peroxidation, while lower H_2_O_2_ concentrations suggest increased scavenging of reactive oxygen species (Gill and Tuteja, 2010). These results demonstrate that AMF support plant growth and play a role in optimizing oxidative metabolism under normal growth conditions. Furthermore, the accumulation of proline is generally a physiological response in plants exposed to various abiotic stresses. In absence of stress, AMF may have little effect on proline concentration, or may even reduce concentration slightly, as the plant does not need to accumulate osmoprotectants such as proline. This was confirmed in CH and TD accessions, for which proline concentrations did not differ between treatments. In contrast, colonized plants from the MJ accession had significantly lower proline concentrations than their non-colonized counterparts. A significant increase in TSS concentration was observed in AMF-colonized plants compared to non-colonized plants for both CH and TD accessions under WW conditions. This suggests that AMF may enhance carbohydrate metabolism even in the absence of drought stress, likely by improving nutrient uptake and photosynthesis activity (Augé, 2001; Ruiz-Lozano, 2003). The increase in TSS concentration could reflect improved carbon allocation or increased transport of sugars from source to sink organs, both have been associated with AMF symbiosis (Smith and Read, 2008). However, no significant differences were observed for the MJ accession, which suggested a possible genotypic response to AMF colonization. Variability in mycorrhizal responsiveness between genotypes has been widely documented (Imaizumi-Ankaru, 2024; Marrassini et al., 2024, 2025) and could be attributed to differences in root architecture, mycorrhizal dependency, or physiological characteristics. The lack of response in the MJ accession suggests that its carbon metabolism under WW conditions may be less influenced by AMF.

### 4.2 AMF mitigates the impact of drought on argan tree accessions

#### 4.2.1 Growth responses of argan accessions under drought stress conditions and role of AMF colonization

Whatever the argan accession, plant growth (i.e., number of leaves, shoot and root length and biomass production) was markedly affected under WS conditions, in the presence as well as absence of AMF colonization. This is consistent with previous studies reporting the detrimental effects of drought stress on the growth of argan trees (Bezzalla et al., 2018). The decrease in biomass can be attributed to a reduction in nutrients uptake. When soil water content is reduced, stomata close, transpiration decreases and, as a result, the flow of water and nutrients slows down due to the reduced rate of diffusion of nutrients through the soil to the root surface (Yoo et al., 2009). The more severe the drought, the lower the flow of water and nutrients and the more limited the availability of nutrients for uptake by the root system. This was emphasized in our study, in which shoot P concentration was significantly lower under WS than WW treatment in non-colonized plants of TD and MJ accessions. Conversely, no significant differences were observed in root P concentration of non-colonized plants across all accessions under WW and WS conditions. This suggests that P accumulates in the roots and is not transferred to the shoots, due to reduced transpiration. Similarly, a significant increase in Na was noticed in the roots of the non-colonized plants under WS compared to WW conditions, likely due to reduced transpiration limiting Na transport to shoots (Ciíaco da Silva et al., 2011). Regarding K, no significant difference was noticed in shoot concentration between non-colonized plants grown under WW and WS conditions, suggesting that K homeostasis was maintained under different water regimes (Wang et al., 2013).

AMF-colonized plants had a significantly higher number of leaves, shoots and roots length, as well as biomass production compared to the non-colonized plants, regardless of accession. Similar observation has been made in argan by Outamamat et al. (2022a, 2022b), Ouallal et al. (2022) and Soufiani et al. (2023) as well as in other plants such as date palm (Baslam et al., 2014; Benhiba et al., 2015), olive tree (Fouad et al., 2014), and carob (Essahibi et al., 2018). This suggest that AMF play a critical role in mitigating the effects of drought stress, possibly by increasing root hydraulic conductivity and nutrient uptake (Sánchez-Romera et al., 2016; Begum et al., 2019).

In roots, particularly in the CH and TD accessions, a significantly higher P concentration was observed in the AMF-colonized plants compared to the respective non-colonized plants. This higher P concentration could be attributed to an increased uptake by the extraradical mycelium of the fungus and possible accumulation in the intraradical structures. AMF improves the acquisition of P by facilitating water transport through its extraradical hyphae, which promotes the mobilization and translocation of inorganic P to the root cortical cells. This process is regulated by the water potential gradient generated by host plant transpiration and the activity of fungal aquaporins. Together, these factors play a critical role in P transport (Kikuchi et al., 2016). In shoot, no significant difference in P concentration was observed between AMF-colonized and non-colonized plants in all accessions, while in the MJ accession, shoot P concentration in AMF-colonized plants was significantly higher in plants under WS than plants under WW conditions. However, shoot P content was significantly higher in AMF-colonized plants compared to non-colonized plants under WS conditions, regardless of the accession (see **Supplementary Results**). This effect could be explained by the increased acquisition of P by the hyphal network, under WS conditions, even in confined spaces such as 1 L pots.

The higher P concentration in AMF-colonized plants versus non-colonized plants under WS conditions can be explained by the unique physiological and structural characteristics of AMF. Their extraradical hyphae can grow and remain active at water potentials much lower than those of roots, allowing them to continue to take up and transport water and nutrients, including P, when root function is slowed down (Augé, 2001; Allen, 2009). Root hairs are more susceptible to desiccation and rapidly lose their efficiency in dry soils (North and Nobel, 1997). In addition, the diameter of hyphae is much smaller than root hairs, enabling them to penetrate tiny soil pores and access water and nutrients that are physically inaccessible to root hairs, particularly during drought when diffusion is limited. This small-scale exploration of the soil increases the effective volume of soil available to the plant for water and P uptake (Jakobsen et al., 1992; Smith and Read, 2008). Under WS conditions, the efficiency of the direct P uptake pathway by roots is often reduced. In contrast, the mycorrhizal pathway is increasingly important, as AMF contribute to maintain nutrient acquisition. This increased contribution of the mycorrhizal pathway promotes P uptake, thus compensating for the reduced functionality of the direct root pathway (Smith and Smith, 2011; Ruiz-Lozano et al., 2016).

The AMF-colonized plants had a significantly higher concentration of K and higher content of K (see **Supplementary Results**) in roots than non-colonized plants, regardless of the accession but also water regime, suggesting that AMF played a beneficial role in enhancing K uptake. This could be attributed to improved transport capacities and soil exploration facilitated by the mycorrhizal network (Garcia and Zimmermann, 2014; Al-Karaki and Williams, 2021). In shoots, no significant difference in K concentration was observed between WS and WW conditions in both AMF-colonized and non-colonized plants. This suggests that K homeostasis was maintained regardless of water availability or mycorrhizal status. As K plays a crucial role in osmoregulation, stomatal opening and enzyme activation, plants may prioritize maintaining its levels during drought stress to preserve essential physiological functions. In some species, K is efficiently remobilized, or its uptake is tightly regulated under stress, resulting in stable tissue concentrations (Wang et al., 2013).

The AMF-colonized plants had a significantly higher K concentration and content (see **Supplementary Results**) in roots than non-colonized plants, regardless of the accession but also WR, suggesting that AMF played a beneficial role in enhancing K uptake. This could be attributed to improved transport capacities and soil exploration facilitated by the mycorrhizal network (Garcia and Zimmermann, 2014; Al-Karaki and Williams, 2021). In shoots, no significant difference in K concentration was observed between WS and WW conditions in both AMF-colonized and non-colonized plants. This suggests that K homeostasis was maintained regardless of water availability or mycorrhizal status. As K plays a crucial role in osmoregulation, stomatal opening and enzyme activation, plants may prioritize maintaining their potassium levels during drought stress to preserve essential physiological functions. In some species, K is efficiently remobilized, or its uptake is tightly regulated under stress, resulting in stable tissue concentrations (Wang et al., 2013).

#### 4.2.2 Physiological responses of argan accessions to drought stress and role of AMF colonization

Whatever the accession, the non-colonized plants grown under WS conditions had significantly lower chlorophyll content than those under WW conditions. This reduction is a typical physiological response to drought, as drought stress disrupts chlorophyll metabolism by increasing chlorophyll degradation and inhibiting the biosynthesis of chlorophyll pigments (Anjum et al., 2011). These disturbances in pigment metabolism reduce the net photosynthetic rates, thereby compromising plant physiological performance under drought conditions (Augé, 2001; Ruiz-Lozano et al., 2016; Bhardwaj et al., 2023; Cui et al., 2024).

Similarly, under WS conditions, the g_s_ of non-colonized plants significantly decreased compared to those under WW conditions. This is a well-known strategy of plants to reduce transpiration and keep water. However, this reduction also limits CO_2_ uptake, leading to decreased photosynthesis and growth (Flexas and Medrano, 2002; Chaves et al., 2009). These results corroborate those of Chakhchar et al. (2016), which reported a decrease of g_s_ in argan trees under water-stressed conditions. Comparable response has been documented in olive trees (El Yamani et al., 2020; Boussadia et al., 2023) and wheat (Ahmad et al., 2018). Finally, the RWC of the non-colonized plants grown under WS conditions was significantly lower than under WW conditions, confirming the findings of Keyvan (2010) and Patanè et al. (2022), which demonstrated that a decrease in RWC under drought stress is associated with reduced water uptake and increased transpiration.

Plant colonization by AMF positively influenced the physiological parameters of argan accessions under WS conditions. Regardless of the accession, AMF-colonized plants had significantly higher RWC, chlorophyll content, and gs than non-inoculated plants. A significant increase in leaf RWC was noticed, indicating improved plant water status under WS conditions. This increase is likely due to the ability of AMF to promote water uptake through, among others, their extensive hyphal network and stimulation of root physiological activity (Wu et al., 2013). By maintaining better hydration, symbiosis with AMF could indirectly promote higher gs and improve photosynthetic performance under WS conditions (Augé, 2001).

The AMF-colonized plants had significantly higher chlorophyll content than non-colonized plants. This increase can be related to several physiological mechanisms. Firstly, AMF increase the uptake of water and nutrients, particularly P, which are vital for the synthesis of chlorophyll and photosynthesis. This was corroborated in our study by a significant increase in P concentration and content in both roots and shoots of AMF-colonized plants, regardless of accession. This increased nutrient availability helps to maintain and synthesize chlorophyll pigments under WS conditions. Similar observations were made in gas exchange parameters, such as gs, between colonized and non-colonized plants. This suggests that AMF colonization could increase photosynthesis by improving the gas exchange capacity of plants under WS conditions. AMF are considered a metabolic sink for the mobilization of photosynthates to plant roots, thereby providing signals for increased photosynthetic activity (Salmeron-Santiago et al., 2021). Numerous studies on the argan tree have shown that AMF-colonization can improve RWC, chlorophyll content and gas exchange (Outamamat et al., 2022a, 2022b; Ouallal et al., 2022).

#### 4.2.3 Biochemical responses of argan accessions to drought stress and role of AMF colonization

Whatever the presence/absence of AMF, WS conditions significantly increased the concentration of H_2_O_2_ and MDA in all accessions compared to the WW conditions. Nonetheless, the impact was significantly more marked in the non-colonized plants, indicating a less pronounced oxidative stress when plants are associated with the AMF. Similar observations have been reported by Fouad et al. (2014) in olive plants and Chitarra et al. (2016) in tomato, which demonstrated that drought stress enhances the production of ROS, including H_2_O_2_. A significantly higher H_2_O_2_ concentration was observed in the CH accession, compared to the MJ and TD accessions, regardless the presence/absence of AMF and water regime, further suggesting a larger oxidative stress and a possible weaker antioxidant defense. This variation in H_2_O_2_ accumulation between accessions may be related to genetic differences in antioxidant defense mechanisms and stress tolerance (Gill and Tuteja, 2010). The colonization of plants with AMF resulted in a significantly lower concentrations of H_2_O_2_ and MDA across all accessions compared to non-colonized plants. The lower MDA concentrations in AMF-colonized plants versus non-colonized plants was already reported by Wu and Xia (2006) in *Poncirus trifoliata citrus tangerine* and Rani et al. (2018) in *Triticum aestivum.* A reduced accumulation of ROS was also reported by Baslam et al. (2014) in *Phoenix dactylifera*, by Fouad et al. (2014) in olive plants and by Benhiba et al. (2015) in date palm, indicating reduced oxidative stress in AMF-colonized plants. Indeed, under drought stress, excessive ROS production can cause cellular damage, especially lipid peroxidation. These observations highlight the protective role of AMF in helping plants maintain cellular balance under drought stress conditions. Among accessions, TD showed the most efficient antioxidant response following AMF colonization (lowest H_2_O_2_), while CH showed the highest oxidative stress indicators. This highlights the importance of genotype in modulating the effectiveness of AMF associations under drought. In addition to this role in mitigating oxidative stress, AMF colonization influences other biochemical responses to drought stress, including the regulation of osmolyte and osmoprotectant accumulation. As such, both TSS and proline concentration increased significantly in the non-colonized plants under WS conditions across all accessions, while AMF-colonized plants maintained stable TSS concentration. This suggests that AMF helps to regulate osmolyte accumulation, by improving water and nutrient uptake such as P and K, particularly observed in MJ and TD accessions, maintaining photosynthetic activity, and minimizing oxidative stress (Ruiz-Sánchez et al., 2010; Barros et al., 2018). These results support the protective role of AMF under drought conditions (Benhiba et al., 2015; Essahibi et al., 2018; Outamamat et al., 2022a, 2022b).

The accumulation of proline is a well-known adaptive response to drought stress and is often considered a reliable biochemical marker of stress resistance in plants (Szabados and Savouré, 2010; Hayat et al., 2012), highlighting its multifaceted role as a key osmoprotectant. Proline contributes to cellular osmotic adjustment by balancing the intracellular osmotic potential, thereby helping cells retain water during dehydration. Proline also plays a critical role in stabilizing proteins, enzymes, and cell membranes, thereby protecting structural integrity under stressful conditions (Szabados and Savouré, 2010; Kavi Kishor and Sreenivasulu, 2014). Moreover, proline acts as a molecular chaperone and an efficient scavenger of ROS, thereby mitigating oxidative damage in stressed tissues (Szabados and Savouré, 2010). The higher proline concentration regardless the accession and the AMF colonization under WS than plant under WW conditions, reflects a well-known plant response to drought stress. This accumulation is widely documented across plant species and is recognized as a reliable biochemical marker of water deficit (Kavi Kishor et al., 2005). The consistency of this response, independent of genotype or symbiotic status, suggests that proline biosynthesis is a fundamental and conserved mechanism of plant stress resistance (Szabados and Savouré, 2010).

## 5 Conclusion

In this study, we demonstrated that AMF significantly improve morphological, physiological, and biochemical traits of argan plants under drought stress conditions. The benefits of AMF colonization were particularly evidenced in the TD and MJ accessions. AMF-colonized plants showed increased shoot and root length, shoot and root biomass, and enhance uptake of both immobile (e.g., P) and mobile (e.g., K) nutrients compared to their respective non-colonized plants. AMF also increased chlorophyll content and stomatal conductance under drought stress conditions. In terms of biochemical traits, AMF colonization reduced oxidative stress by lowering the accumulation of MDA and H_2_O_2_ and modulating the accumulation of proline and TSS.

The three argan accessions responded differently to colonization by *R. irregularis* under drought stress conditions. Mejii accession showed a positive response to drought stress, demonstrating significant improvements in growth, nutrient uptake, and stomatal conductance, as well as reduced oxidative stress. Tidzi accession also performed well, showing increased biomass and nutrient uptake, as well as reduced oxidative damage. In contrast, City Hanchan accession showed some growth improvements, but also showed higher H_2_O_2_ concentrations, indicating higher sensitivity to drought stress. Overall, MJ and TD accessions exhibited a significantly enhanced responses to mycorrhization under drought stress compared to CH accession. Performing transcriptome analyses would strengthen our understanding on the variability of responses of the argan accessions, revealing some of the mechanisms involved in the increased resistance of AMF-colonized argan trees to drought stress.

This study highlights the genotypic variability of argan accessions in their response to AMF colonization, which has important implications for breeding programs aimed at improving drought resistance. This variability suggests that the selection and propagation of AMF-compatible genotypes could be a promising strategy to improve the adaptability of argan trees to climate change. However, the results are based on controlled pot experiments that do not fully consider the complexity of natural soil and field conditions. Pot experiments may exaggerate root confinement, alter soil microbial interactions, and limit long-term plant-fungus dynamics. Therefore, future research should prioritize field trials in diverse agroecological zones to validate these findings and better understand the stability of AMF benefits under varying environmental conditions. Integrating AMF inoculation strategies into argan breeding programs could represent a sustainable approach to improve the resilience and productivity of this species in regions subject to drought.

## Data Availability

The original contributions presented in the study are included in the article/**Supplementary Material**. Further inquiries can be directed to the corresponding author.

## Footnotes

1 Glomeromycota IN vitro Collection, BCCM-MUCL, http://www.mycorrhiza.be/ginco-bel

2
https://bccm.belspo.be/about-MUCL
