# Correction: Drought resistance of *Argania spinosa* L. colonized by the arbuscular mycorrhizal fungus *Rhizophagus irregularis* varies according to accession

**DOI:** 10.3389/fpls.2025.1730846

**Published:** 2025-11-19

**Authors:** Frontiers Production Office

**Affiliations:** Frontiers Media SA, Lausanne, Switzerland

**Keywords:** *argania spinosa*, arbuscular mycorrhizal fungi, water stress, phosphorus uptake, antioxidant metabolism, accession variability

## Abstract

**Figure:**
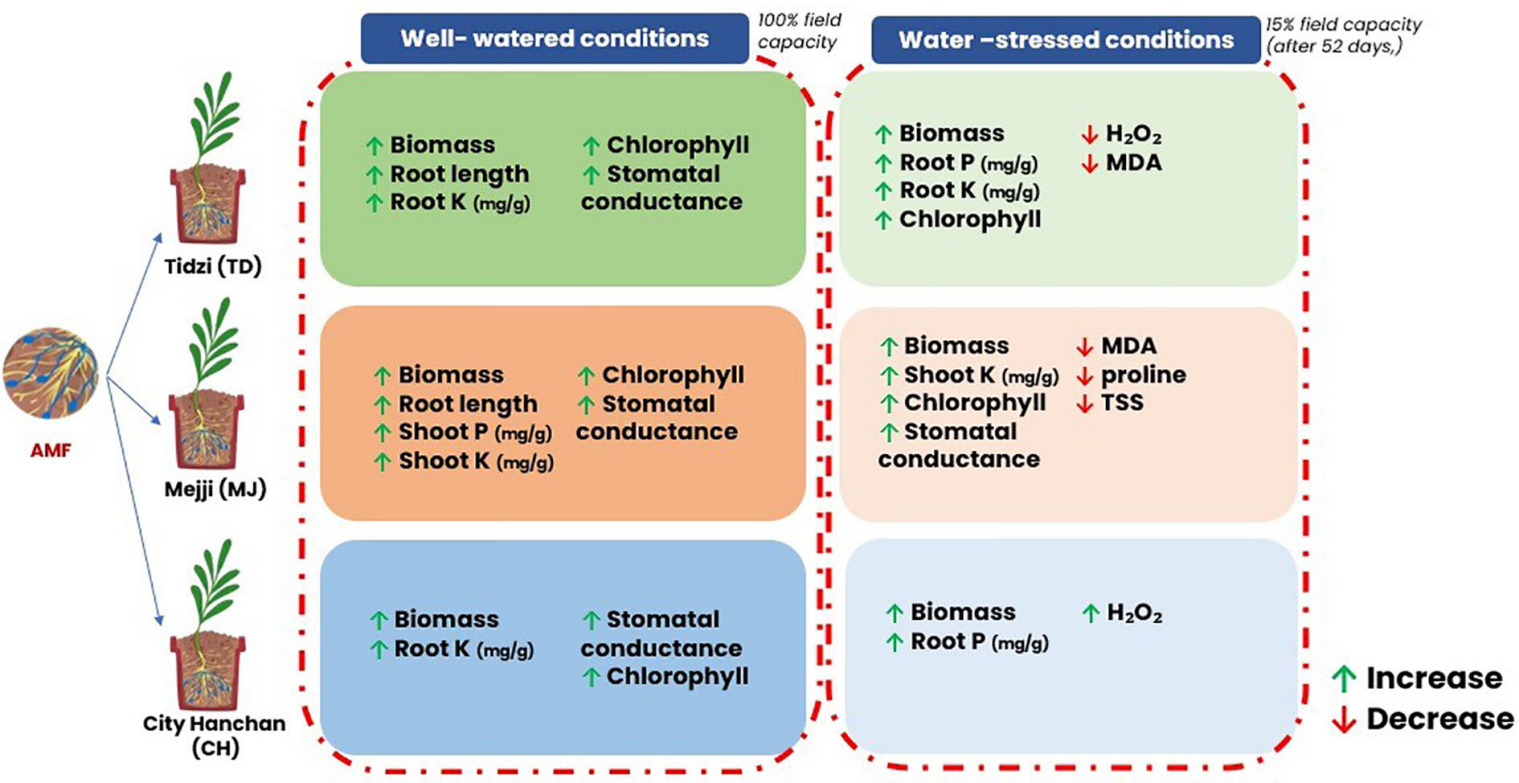

The “Graphical Abstract” was incorrectly labeled as **Figure 6** online and incorrectly shown as **Figure 1** within the PDF file.

The original version of this article has been updated.

