# Supplementary material for: Drought resistance of Argania spinosa L. colonized by the arbuscular mycorrhizal fungus Rhizophagus irregularis varies according to accession
Source: Front Plant Sci. 2025 Oct 15;16:1678553. doi: 10.3389/fpls.2025.1678553 (PMC12568639; doi:10.3389/fpls.2025.1678553)
Supplement: Supplementary file 1 [file DataSheet1.docx]

Supplementary Material

**Table 1.** Effects of accessions (CH, MJ, TD), water regimes (WW and WS) and AMF colonization (NM and M) by *R. irregularis* MUCL 41833 on the shoot and root lengths of argan plants after 52 weeks of stress conditions.

|  | **Shoot length (cm)** | **Root length (cm)** |
| --- | --- | --- |
| **Effect A** | | |
| CH | 18.38 ± 4.84 | 30.75 ± 8.83 |
| MJ | 21.42 ± 6.33 | 33.50 ± 12.61 |
| TD | 19.96 ± 4.55 | 27.08 ± 9.23 |
| **Effect AMF** | | |
| NM | 16.33 ± 4.81 | 23.58 ± 9.20 |
| M | 23.50 ± 2.71 | 37.31 ± 6.22 |
| **Effect WR** | | |
| WW | 21.64 ± 3.99 | 33.58 ± 7.23 |
| WS | 18.19 ± 5.95 | 27.31 ± 12.24 |
| **Effect A * AMF** | | |
| CH/NM | 14.83 ± 3.87 | 24.58 ± 8.55**^C^** |
| CH/M | 21.92 ± 2.54 | 36.92 ± 2.69**^AB^** |
| MJ/NM | 17.17 ± 6.34 | 23.08 ± 8.79**^C^** |
| MJ/M | 25.67 ± 2.16 | 43.92 ± 3.47**^A^** |
| TD/NM | 17.00 ± 4.43 | 23.08 ± 11.65**^C^** |
| TD/M | 22.92 ± 2.20 | 31.08 ± 3.64**^BC^** |
| **Effect A * WR** | | |
| CH/WW | 19.58 ± 2.87 | 33.50 ± 5.01 |
| CH/WS | 17.17 ± 6.31 | 28.00 ± 11.33 |
| MJ/WW | 24.00 ± 4.24 | 36.75 ± 7.43 |
| MJ/WS | 18.83 ± 7.36 | 30.25 ± 16.41 |
| TD/WW | 21.33 ± 4.03 | 30.50 ± 8.61 |
| TD/WS | 18.58 ± 4.96 | 23.67 ± 9.22 |
| **Effect AMF * WR** | | |
| NM/WW | 19.67 ± 4.06**^a^** | 30.33 ± 7.36**^b^** |
| M/WW | 23.61 ± 2.96**^b^** | 16.83 ± 4.81**^ab^** |
| NM/WS | 13.00 ± 2.78**^a^** | 36.83 ± 5.77**^c^** |
| M/WS | 23.39 ± 2.62**^a^** | 37.78 ± 6.96**^a^** |
| **Effect A * AMF * WR** | | |
| CH/NM/WW | 17.67 ± 2.52 | 30.67 ± 5.69 |
| CH/NM/WS | 12.00 ± 2.65 | 18.50 ± 6.26 |
| CH/M/WW | 21.50 ± 1.80 | 36.33 ± 2.52 |
| CH/M/WS | 22.33 ± 3.51 | 37.50 ± 3.28 |
| MJ/NM/WW | 21.67 ± 4.73 | 30.50 ± 2.78 |
| MJ/NM/WS | 12.67 ± 4.16 | 15.67 ± 4.51 |
| MJ/M/WW | 26.33 ± 2.52 | 43.00 ± 3.61 |
| MJ/M/WS | 25.00 ± 2.00 | 40.83 ± 3.82 |
| TD/NM/WW | 19.67 ± 5.03 | 29.83 ± 13.27 |
| TD/NM/WS | 14.33 ± 1.53 | 16.33 ± 5.13 |
| TD/M/WW | 23.00 ± 2.65 | 31.17 ± 2.84 |
| TD/M/WS | 22.83 ± 2.25 | 31.00 ± 5.00 |
| **p-value** | | |
| Effect A | 0.080 | **0.033** |
| Effect AMF | **0.000** | **0.000** |
| Effect WR | **0.003** | **0.003** |
| Effect A * AMF | 0.608 | **0.031** |
| Effect A * WR | 0.515 | 0.955 |
| Effect AMF * WR | **0.005** | **0.001** |
| Effect A * AMF * WR | 0.889 | 0.916 |

Data are presented as means +/- SD. Data were analyzed by a three-way ANOVA followed by contrasts post-hoc test (p<0.05) for normalized data. For non-normalized data, a Kruskall-Wallis test was applied. For each effect in a column, different lower-case letters indicate significant difference among the treatments. The absence of any sign indicates no difference among the treatments.

**Table 2.** Effects of accessions (CH, MJ, TD), water regimes (WW and WS) and AMF colonization (NM and M) by *R. irregularis* MUCL 41833 on the K and Na concentrations on roots and on the k concentrations on shoots of argan plants after 52 weeks of stress conditions.

|  | **Roots** | | **Shoots** |
| --- | --- | --- | --- |
|  | **K (mg/g)** | **Na (mg/g)** | **K (mg/g)** |
| **Effect A** | | | |
| CH | 1.42 ± 0.42 | 6.742 ± 2.38 | 1.11 ± 0.13 |
| MJ | 1.64 ± 0.28 | 8.69 ± 4.52 | 1.14 ± 0.39 |
| TD | 1.24 ± 0.39 | 7.6 ± 2.62 | 1.08 ± 0.22 |
| **Effect AMF** | | | |
| NM | 1.22 ± 0.31**^B^** | 9.42 ± 3.05 | 1.22 ± 0.29 |
| M | 1.65 ± 0.35**^A^** | 5.93 ± 2.63 | 1.22 ± 0.29 |
| **Effect WR** | | | |
| WW | 1.29 ± 0.37 | 7.09 ± 3.99**^α^** | 1.14 ± 0.34 |
| WS | 1.57 ± 0.36 | 8.26 ± 2.46**^β^** | 1.07 ± 0.16 |
| **Effect A * AMF** | | | |
| CH/NM | 1.13 ± 0.11 | 8.35 ± 2.22 | 1.18 ± 0.08 |
| CH/M | 1.71 ± 0.41 | 5.13 ± 1.16 | 1.03 ± 0.14 |
| MJ/NM | 1.52 ± 0.28 | 12.45 ± 3.00 | 1.35 ± 0.39 |
| MJ/M | 1.75 ± 0. 25 | 4.93 ± 1.46 | 0.93 ± 0.29 |
| TD/NM | 0.99 ± 0.22 | 7.47 ± 0.91 | 1.14 ± 0.30 |
| TD/M | 1.49 ± 0.38 | 7.73 ± 3.77 | 1.01 ± 0.09 |
| **Effect A * WR** | | | |
| CH/WW | 1.28 ± 0.28 | 5.78 ± 1.40 | 1.05 ± 0.17 |
| CH/WS | 1.56 ± 0.50 | 7.70 ± 2.88 | 1.17 ± 0.03 |
| MJ/WW | 1.60 ± 0.37 | 9.35 ± 6.21 | 1.21 ± 0.55 |
| MJ/WS | 1.67 ± 0.18 | 8.03 ± 2.32 | 1.07 ± 0.17 |
| TD/WW | 1.00 ± 0.22 | 6.15 ± 2.08 | 1.18 ± 0.21 |
| TD/WS | 1.48 ± 0.38 | 9.05 ± 2.39 | 0.98 ± 0.20 |
| **Effect AMF * WR** | | | |
| NM/WW | 1.06 ± 0.23 | 9.71 ± 4.11 | 1.41 ± 0.24**^a^** |
| M/WW | 1.37 ± 0.31 | 9.13 ± 1.65 | 1.04 ± 0.19**^b^** |
| NM/WS | 1.52 ± 0.35 | 4.48 ± 1.24 | 0.90 ± 0.16**^ab^** |
| M/WS | 1.77 ± 0.32 | 7.39 ± 2.90 | 1.10 ± 0.1**^ab^** |
| **Effect A * AMF * WR** | | | |
| CH/NM/WW | 1.04 ± 0.42 | 6.50 ± 1.41 | 1.18 ± 0.13 |
| CH/NM/WS | 1.21 ± 0.09 | 10.20 ± 0.20 | 1.18 ± 0.26 |
| CH/M/WW | 1.51 ± 0.18 | 5.07 ± 1.18 | 0.91 ± 0.29 |
| CH/M/WS | 1.90 ± 0.51 | 5.20 ± 1.39 | 1.15 ± 0.21 |
| MJ/NM/WW | 1.29 ± 0.18 | 14.80 ± 2.34 | 1.70 ± 0.09 |
| MJ/NM/WS | 1.75 ± 0.05 | 10.10 ± 0.66 | 0.99 ± 0.07 |
| MJ/M/WW | 1.91 ± 0.13 | 3.90 ± 1.39 | 0.71 ± 0.13 |
| MJ/M/WS | 1.59 ± 0.25 | 5.97 ± 0.45 | 1.15 ± 0.23 |
| TD/NM/WW | 0.85 ± 0.16 | 7.83 ± 0.76 | 1.35 ± 0.15 |
| TD/NM/WS | 1.15 ± 0.17 | 7.10 ± 1.04 | 0.94 ± 0.29 |
| TD/M/WW | 1.16 ± 0.14 | 4.47 ± 1.33 | 1.01 ± 0. 10 |
| TD/M/WS | 1.82 ± 0.08 | 11.00 ± 1.32 | 1.00 ± 0.1.05 |
| **p-value** | | | |
| Effect A | 0.527 | **0.021** | 0.078 |
| Effect AMF | **0.007** | 0.255 | 0.084 |
| Effect WR | 0.074 | **0.000** | 0.739 |
| Effect A * AMF | 0.075 | **0.001** | **0.000** |
| Effect A * WR | 0.260 | 0.177 | **0.028** |
| Effect AMF * WR | 0.977 | 0.690 | **0.042** |
| Effect A * AMF * WR | 0.540 | 0.770 | 0.357 |

Data are presented as means +/- SD. Data were analyzed by a three-way ANOVA followed by contrasts post-hoc test (p<0.05) for normalized data. For non-normalized data, a Kruskall-Wallis test was applied. For each effect in a column, different lower-case letters indicate significant difference among the treatments. The absence of any sign indicates no difference among the treatments.

**Table 3.** Effects of accessions (CH, MJ, TD), water regimes (WW and WS) and AMF colonization (NM and M) by *R. irregularis* MUCL 41833 on the gs and RWC of argan plants after 52 weeks of stress conditions.

|  | **gs (mmol m^-2^ s^-1^)** | **RWC (%)** |
| --- | --- | --- |
| **Effect A** | | |
| CH | 208.90 ± 73.60 | 82.61 ± 8.55 |
| MJ | 199.30 ± 57.50 | 84.11 ± 9.49 |
| TD | 211.30 ± 69.20 | 86.03 ± 10.56 |
| **Effect AMF** | | |
| NM | 150.42 ± 38.73 | 80.16 ± 11.67 |
| M | 262.52 ± 25.47 | 88.34 ± 3.21 |
| **Effect WR** | | |
| WW | 235.50 ± 50.80 | 89.60 ± 3.70 |
| WS | 177.40 ± 66.60 | 78.90 ± 10.38 |
| **Effect A * AMF** | | |
| CH/NM | 145.90 ± 44.30 | 78.11 ± 10.29 |
| CH/M | 271.82 ± 21.29 | 87.11 ± 2.54 |
| MJ/NM | 151.20 ± 39.20 | 80.49 ± 12.54 |
| MJ/M | 247.37 ± 13.74 | 87.73 ± 3.07 |
| TD/NM | 154.10 ± 39.50 | 81.87 ± 13.83 |
| TD/M | 268.40 ± 33.80 | 90.19 ± 3.59 |
| **Effect A * WR** | | |
| CH/WW | 238.70 ± 57.50 | 92.06 ± 3.13 |
| CH/WS | 179.10 ± 80.50 | 80.00 ± 12.18 |
| MJ/WW | 223.20 ± 39.90 | 88.72 ± 4.41 |
| MJ/WS | 175.30 ± 65.60 | 79.50 ± 11.31 |
| TD/WW | 244.70 ± 59.70 | 88.03 ± 2.48 |
| TD/WS | 177.80 ± 65.40 | 77.20 ± 9.19 |
| **Effect AMF * WR** | | |
| NM/WW | 187.78 ± 2.88 | 90.72 ± 4.24 |
| M/WW | 113.06 ± 6.27 | 69.59 ± 4.50 |
| NM/WS | 283.28 ± 18.38 | 88.48 ± 2.87 |
| M/WS | 241.76 ± 8.40 | 88.21 ± 3.70 |
| **Effect A * AMF * WR** | | |
| CH/NM/WW | 187.78 ± 5.36 | 88.97 ± 2.25 |
| CH/NM/WS | 103.98 ± 4.93 | 85.25 ± 0.83 |
| CH/M/WW | 185.13 ± 4.43 | 87.08 ± 2.75 |
| CH/M/WS | 124.88 ± 2.68 | 69.14 ± 3.97 |
| MJ/NM/WW | 184.92 ± 3.29 | 86.16 ± 3.80 |
| MJ/NM/WS | 114.93 ± 2.95 | 89.31 ± 1.32 |
| MJ/M/WW | 191.48 ± 4.32 | 91.28 ± 3.80 |
| MJ/M/WS | 132.58 ± 3.30 | 69.70 ± 5.42 |
| TD/NM/WW | 191.22 ± 4.26 | 93.81 ± 3.91 |
| TD/NM/WS | 120.17 ± 2.60 | 90.06 ± 5.67 |
| TD/M/WW | 202.62 ± 4.35 | 90.31 ± 0.12 |
| TD/M/WS | 144.80 ± 3.49 | 69.94 ± 5.94 |
| **z-value** | | |
| Effect A | 0.318 | 0.258 |
| Effect AMF | **0.004** | 0.066 |
| Effect WR | **0.000** | **0.002** |
| Effect A * AMF |  |  |
| Effect A * WR |  |  |
| Effect AMF * WR |  |  |
| Effect A * AMF * WR |  |  |

Data are presented as means +/- SD. Data were analyzed by a three-way ANOVA followed by contrasts post-hoc test (p<0.05) for normalized data. For non-normalized data, a Kruskall-Wallis test was applied. For each effect in a column, different lower-case letters indicate significant difference among the treatments. The absence of any sign indicates no difference among the treatments.

**Table 4.** Effects of accessions (CH, MJ, TD), water regimes (WW and WS) and AMF colonization (NM and M) by *R. irregularis* MUCL 41833 on the proline and H_2_O_2_ concentrations of argan plants after 52 weeks of stress conditions.

|  | **Proline (µmol/g)** | **H_2_O_2_ (µmol/g)** |
| --- | --- | --- |
| **Effect A** | | |
| CH | 5.23 ± 1.28 | 2.67 ± 1.51**^a^** |
| MJ | 2.58 ± 1.33 | 1.94 ± 0.94**^b^** |
| TD | 2.15 ± 1.14 | 1.56 ± 0.75**^c^** |
| **Effect AMF** | | |
| NM | 3.78 ± 1.66 | 2.39 ± 1.34 **^β^** |
| M | 2.86 ± 1.94 | 1.73 ± 0.91**^α^** |
| **Effect WR** | | |
| WW | 2.40 ± 1.57**^b^** | 1.13 ± 0.27 |
| WS | 4.24 ± 1.64**^a^** | 2.98 ± 0.99 |
| **Effect A * AMF** | | |
| CH/NM | 5.32 ± 1.31 | 3.13 ± 1.79 |
| CH/M | 5.13 ± 1.37 | 2.21 ± 1.15 |
| MJ/NM | 3.37 ± 1.18 | 2.24 ± 1.06 |
| MJ/M | 1.80 ± 1.00 | 1.65 ± 0.78 |
| TD/NM | 2.65 ± 1.29 | 1.79 ± 0.83 |
| TD/M | 1.64 ± 0.76 | 1.32 ± 0.64 |
| **Effect A * WR** | | |
| CH/WW | 4.36 ± 0.79 | 1.34 ± 0.29 |
| CH/WS | 6.09 ± 1.09 | 4.00 ± 0.85 |
| MJ/WW | 1.61 ± 0.81 | 1.12 ± 0.20 |
| MJ/WS | 3.55 ± 0.98 | 2.77 ± 0.53 |
| TD/WW | 1.23 ± 0.36 | 0.93 ± 0.15 |
| TD/WS | 3.07 ± 0.82 | 2.19 ± 0.51 |
| **Effect AMF * WR** | | |
| NM/WW | 2.88 ± 1.57 | 1.29 ± 0.23 |
| M/WW | 4.68 ± 1.24 | 3.49 ± 1.03 |
| NM/WS | 1.92 ± 1.50 | 0.98 ± 0.23 |
| M/WS | 3.79 ± 1.93 | 2.48 ± 0.66 |
| **Effect A * AMF * WR** | | |
| CH/NM/WW | 4.81 ± 0.93 | 1.50 ± 0.23 |
| CH/NM/WS | 5.83 ± 1.62 | 4.76 ± 0.12 |
| CH/M/WW | 3.91 ± 0.30 | 1.19 ± 0.29 |
| CH/M/WS | 6.35 ± 0.70 | 3.24 ± 0.23 |
| MJ/NM/WW | 2.33 ± 0.25 | 1.30 ± 0.06 |
| MJ/NM/WS | 4.40 ± 0.45 | 3.17 ± 0.43 |
| MJ/M/WW | 0.89 ± 0.05 | 0.95 ± 0.04 |
| MJ/M/WS | 2.70 ± 0.11 | 2.36 ± 0.16 |
| TD/NM/WW | 1.50 ± 0.30 | 1.06 ± 0.06 |
| TD/NM/WS | 3.80 ± 0.26 | 2.53 ± 0.35 |
| TD/M/WW | 0.95 ± 0.02 | 0.79 ± 0.02 |
| TD/M/WS | 2.33 ± 0.08 | 1.85 ± 0.42 |
| **p-value** | | |
| Effect A | 0.000 | **0.000** |
| Effect AMF | 0.000 | **0.000** |
| Effect WR | **0.000** | **0.000** |
| Effect A * AMF | **0.028** | 0.997 |
| Effect A * WR | 0.907 | 0.067 |
| Effect AMF * WR | 0.847 | 0.548 |
| Effect A * AMF * WR | 0.063 | 0.707 |

Data are presented as means +/- SD. Data were analyzed by a three-way ANOVA followed by contrasts post-hoc test (p<0.05) for normalized data. For non-normalized data, a Kruskall-Wallis test was applied. For each effect in a column, different lower-case letters indicate significant difference among the treatments. The absence of any sign indicates no difference among the treatments.

**A**

**B**


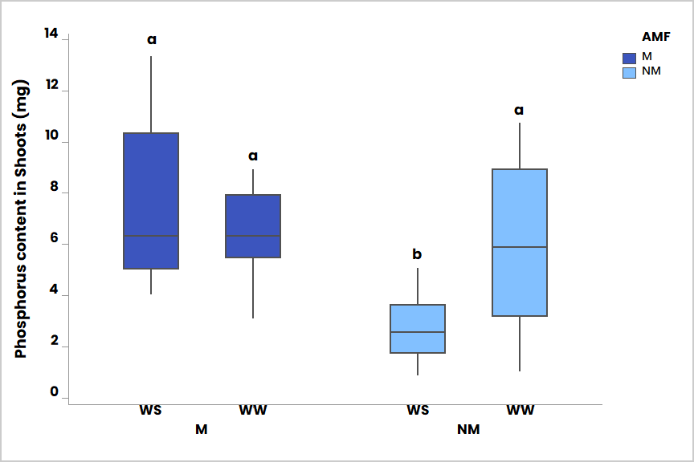

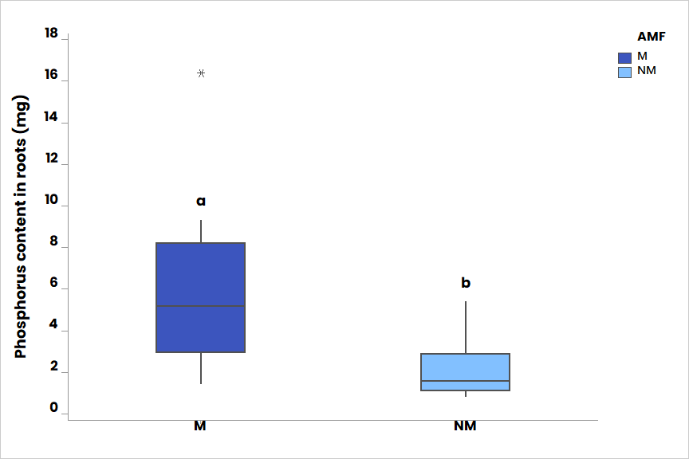


**C**


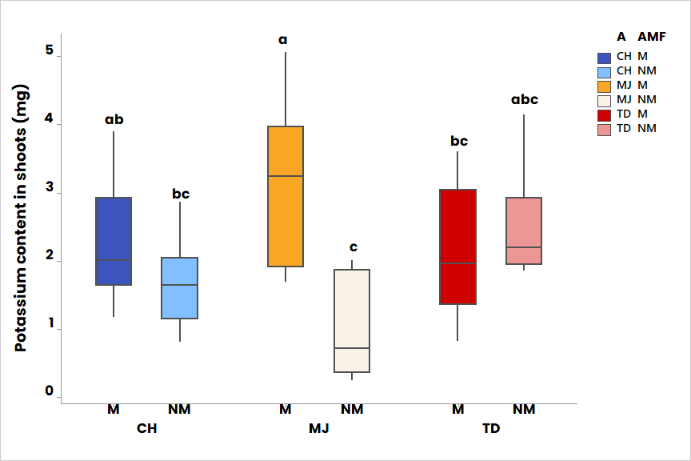

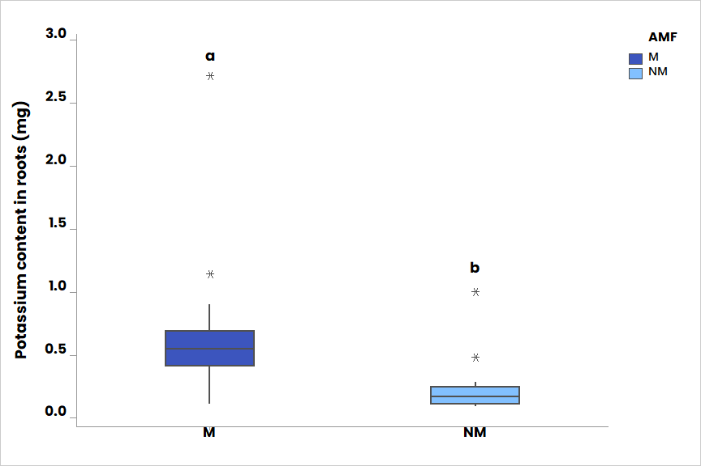


**D**

**E**


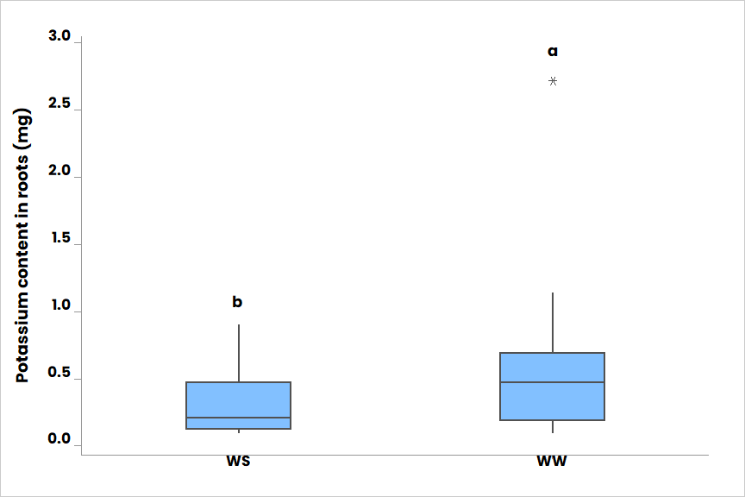


**Figure 1**. Effect of water regime (WR) (i.e., 100% of field capacity = Well-Watered (WW) and 15% of field capacity = Water-Stressed (WS)), AMF colonization by *Rhizophagus irregularis* MUCL 41833 (i.e. mycorrhizal (M) or non-mycorrhizal (NM)) and accession (i.e., TD (Tidzi), MJ (Mejji), and CH (City El Hanchan)) on nutrients content at harvest (52 days after the start of the WR application). Significance of (A) AMF and WR interaction on phosphorus content in shoots, (B) AMF on phosphorus content in roots, and AMF and accession interaction, (C) accession and AMF interactions on potassium content in shoots and (D) AMF on potassium content in roots, (E) WR on potassium content in roots. The box plots display the maximum, upper quartile, and lower quartile minimum values. Data were analyzed by a three-way ANOVA followed by a Tukey post-hoc test (P ≤ 0.05). For one parameter, data sharing similar lower-case letters are not significantly different.


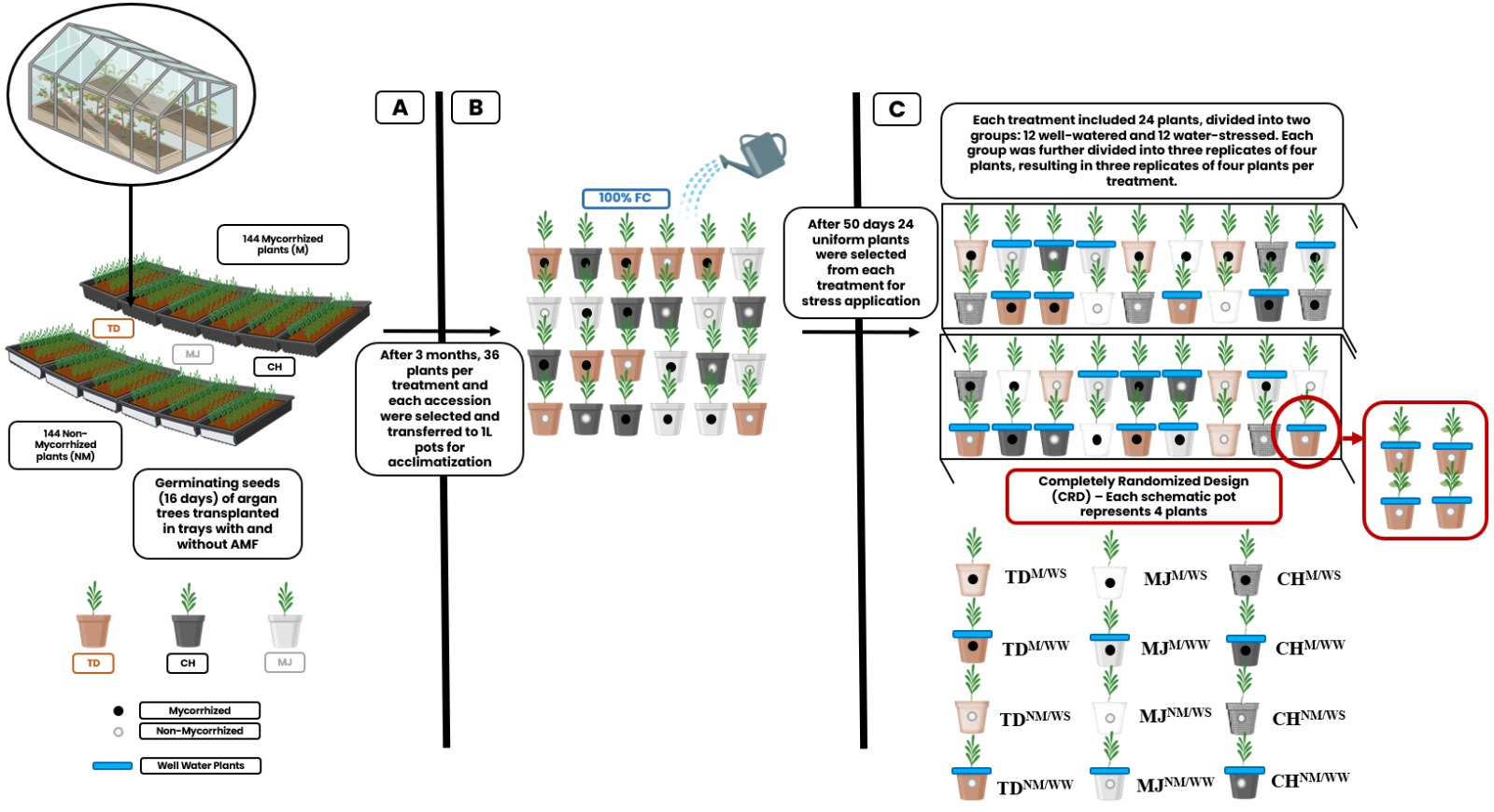


**Figure 2**. Experimental set up to evaluate the effects of mycorrhization on three argan accession grown under two water regimes.
